# A Large Dengue Outbreak in Taiwan, 2023: Driven by Imported Cases, Serotype Cocirculation, and Climate Variability

**DOI:** 10.1093/ofid/ofag070

**Published:** 2026-02-21

**Authors:** Jie-Yu Huang, Shih-Feng Weng, Zih-Syuan Yang, Ying-Wei Tung, Wen-Hung Wang, Wanchai Assavalapsakul, Arunee Thitithanyanont, Day-Yu Chao, Chun-Yu Lin, Yen-Hsu Chen, Sheng-Fan Wang

**Affiliations:** Center for Tropical Medicine and Infectious Disease, Kaohsiung Medical University, Kaohsiung, Taiwan; Department of Medical Laboratory Science and Biotechnology, Kaohsiung Medical University, Kaohsiung, Taiwan; Department of Healthcare Administration and Medical Informatics, College of Health Sciences, Kaohsiung Medical University, Kaohsiung, Taiwan; Center for Big Data Research, Kaohsiung Medical University, Kaohsiung, Taiwan; Center for Tropical Medicine and Infectious Disease, Kaohsiung Medical University, Kaohsiung, Taiwan; Department of Medical Laboratory Science and Biotechnology, Kaohsiung Medical University, Kaohsiung, Taiwan; Center for Tropical Medicine and Infectious Disease, Kaohsiung Medical University, Kaohsiung, Taiwan; Department of Medical Laboratory Science and Biotechnology, Kaohsiung Medical University, Kaohsiung, Taiwan; Center for Tropical Medicine and Infectious Disease, Kaohsiung Medical University, Kaohsiung, Taiwan; School of Medicine, College of Medicine, National Sun Yat-Sen University, Kaohsiung, Taiwan; Department of Microbiology, Faculty of Science, Chulalongkorn University, Bangkok, Thailand; Department of Microbiology, Faculty of Science, Mahidol University, Bangkok, Thailand; Graduate Institute of Microbiology and Public Health, College of Veterinary Medicine, National Chung Hsing University, Taichung, Taiwan; Center for Tropical Medicine and Infectious Disease, Kaohsiung Medical University, Kaohsiung, Taiwan; Division of Infectious Disease, Department of Internal Medicine, Kaohsiung Medical University Hospital, Kaohsiung, Taiwan; Center for Tropical Medicine and Infectious Disease, Kaohsiung Medical University, Kaohsiung, Taiwan; School of Medicine, College of Medicine, National Sun Yat-Sen University, Kaohsiung, Taiwan; Division of Infectious Disease, Department of Internal Medicine, Kaohsiung Medical University Hospital, Kaohsiung, Taiwan; Center for Tropical Medicine and Infectious Disease, Kaohsiung Medical University, Kaohsiung, Taiwan; Department of Medical Laboratory Science and Biotechnology, Kaohsiung Medical University, Kaohsiung, Taiwan; Department of Medical Research, Kaohsiung Medical University Hospital, Kaohsiung, Taiwan; M.Sc. Program in Tropical Medicine, College of Medicine, Kaohsiung Medical University, Kaohsiung, Taiwan

**Keywords:** climate factors, dengue outbreak, imported cases, Taiwan, time-lag analysis

## Abstract

**Background:**

Taiwan, a region traditionally considered non-endemic for dengue, experienced an unexpected and large-scale outbreak in 2023. We investigated the multifactorial drivers of this outbreak, including cross-border viral importation, serotype cocirculation, vector ecology, and climate variability.

**Methods:**

We analyzed national dengue surveillance data (2013–2023), meteorological records, and Breteau Index (BI) values, alongside molecular serotyping and whole-genome sequencing of clinical isolates. Time-lagged Poisson regression was used to identify predictors of indigenous dengue transmission in Kaohsiung and Tainan. Full-genome comparisons were conducted between 2023 strains and historical epidemic isolates.

**Results:**

A total of 26 706 laboratory-confirmed cases were reported, primarily in Tainan (80.7%) and Kaohsiung (11.9%). Real-time RT-PCR identified cocirculating DENV-1 and DENV-2 strains. Phylogenetic analysis confirmed the 2023 DENV-1 and DENV-2 strains were genetically linked to contemporary strains from Southeast Asian countries. Whole-genome sequencing identified several nonsynonymous mutations in the NS2A, NS3, and NS5 regions when compared with historical outbreak isolates. Time-lagged regression showed that imported cases, precipitation, and the BI were associated with incidence in univariate models. In Kaohsiung, the best-fitting multivariable model included the BI, but temperature and precipitation were the independent predictors. In Tainan, precipitation and, at longer lags, imported cases were more influential, while the BI lost significance after adjustment.

**Conclusions:**

The 2023 dengue outbreak in Taiwan was driven by a complex interplay between viral introductions, climatic conditions, and vector dynamics. The differing transmission drivers observed between cities highlight the need for region-specific vector surveillance, climate-informed early warning systems, and sustained genomic monitoring to prevent future re-emergence of dengue in this non-endemic setting.

## Teaser

An unexpected large dengue outbreak occurred in Taiwan in 2023, driven by imported cases, serotype cocirculation, climate variation, and vector density. Region-specific time-lagged models identified differing transmission drivers between Kaohsiung and Tainan, highlighting the need for targeted control strategies.

## Graphical Abstract

**Graphical Abstract:**
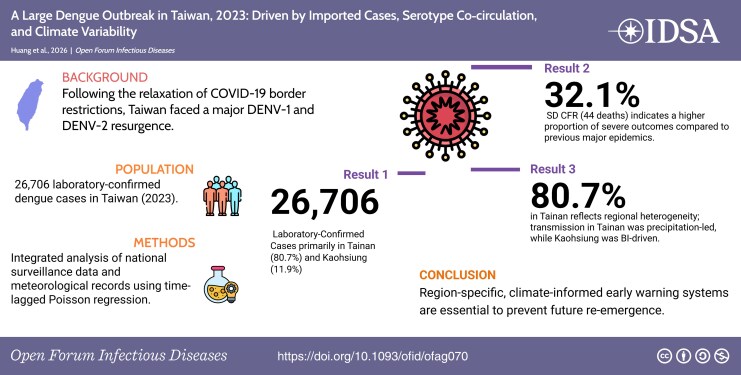
This graphical abstract is also available at Tidbit: https://tidbitapp.io/tidbits/a-large-dengue-outbreak-in-taiwan-2023-driven-by-imported-cases-serotype-co-circulation-and-climate-variability-cf1fdbc5-bd6f-4791-8066-5ca1117fcab7

Highlights

1. Taiwan experienced a major dengue resurgence in 2023 following post–COVID-19 border reopening.
2. DENV-1 and DENV-2 cocirculated, and phylogenetic analysis showed close relatedness to Southeast Asian strains.
3. Imported cases, rainfall, and the Breteau Index (BI) were key predictors in time-lagged regression models.
4. Stronger BI–case associations were observed in Kaohsiung, whereas Tainan showed greater sensitivity to climatic factors.

Dengue virus (DENV) is a mosquito-borne, positive-sense RNA virus classified within the genus *Orthoflavivirus*, family Flaviviridae [1, 2]. It is a leading cause of febrile illness in tropical and subtropical regions, placing approximately 3.9 billion people at risk globally [3–5]. The primary vector is *Aedes aegypti*, with *Aedes albopictus* serving as a secondary vector [6, 7]. DENV comprises 4 antigenically distinct serotypes (DENV-1 to DENV-4), all capable of causing a spectrum of illness, ranging from asymptomatic infection to severe dengue with hemorrhagic manifestations and organ involvement [8].

Taiwan, an island nation in the western Pacific, experiences a climate ranging from subtropical to tropical. This climate creates an ideal environment for the proliferation and survival of *Aedes* mosquitoes. While *A. albopictus* is ubiquitous across the island, *A. aegypti* populations are predominantly found in the southern regions [9–12]. Dengue epidemics have been a recurring issue in Taiwan since the late 1800s and continue to present a considerable public health challenge [13–15].

In recent years, the COVID-19 pandemic significantly influenced global infectious disease trends, largely due to strict travel restrictions and public health interventions. During the period from 2020 to 2022, Taiwan observed a marked reduction in dengue activity, coinciding with stringent border controls and decreased international travel [16]. However, after the relaxation of border restrictions in late 2022, Taiwan witnessed a sharp rise in dengue cases, leading to a widespread outbreak in 2023. This represented the third major dengue epidemic within a decade, following significant outbreaks in 2014 and 2015, with the southern regions once again bearing the greatest burden [17].

Although Taiwan is not traditionally considered as a dengue-endemic region, there are annual outbreaks linked to imported cases [18–20]. Nevertheless, in addition to viral importation, environmental suitability and climatic conditions are recognized as key contributors to local transmission dynamics [21, 22]. The resurgence of dengue likely reflects the interplay of multiple factors, including altered population immunity, changes in vector ecology, increased international mobility, and possible importation of novel viral strains from endemic regions in neighboring countries. Despite these associations, the precise drivers behind the 2023 outbreak remain to be fully elucidated. In this study, we aimed to elucidate the multifactorial drivers of the 2023 dengue resurgence in Taiwan. Specifically, we assessed the relative contributions of imported cases, serotype distribution, vector indices, and meteorological conditions to local transmission patterns, using integrated epidemiological, genomic, and ecological data.

## MATERIALS AND METHODS

### Data Sources

Monthly dengue case counts (imported and indigenous) for 2013–2024 were obtained from the Taiwan Centers for Disease Control (Taiwan CDC) surveillance database (https://www.cdc.gov.tw).

The Breteau Index (BI), defined as the number of larval-positive containers per 100 inspected households, is routinely monitored by local health authorities under the supervision of the Taiwan CDC. This vector surveillance is conducted at the neighborhood (Li, or village) level. Analysis of the raw surveillance data shows that inspections are reported daily, and a standard inspection event typically covers approximately 50 households (Data Median = 50; 75th percentile = 52) per Li. (Optional: This aligns with national CDC guidelines, which recommend ≥20% Li coverage during June–November and ≥10% during December–May.)

For this study, we obtained the raw daily vector surveillance reports from the Taiwan CDC Open Data Platform. To match the temporal resolution of the dengue case and meteorological datasets, these daily reports were re-aggregated to calculate a single, weighted monthly BI. This was achieved by summing the total number of larval-positive containers (PosConAll) and the total number of households inspected (HouseHold) from all reports within a calendar month, and then applying the standard BI formula. This method ensures that all inspections are appropriately weighted by their sample size and avoids the bias of averaging precalculated indices.

Meteorological variables, including average temperature and total monthly precipitation, were retrieved from the Central Weather Administration open-data portal (https://www.cwa.gov.tw).

All datasets were stratified by city (Tainan and Kaohsiung) and calendar month. Taiwan CDC datasets were used for descriptive epidemiological analyses and time-lagged regression modeling, while clinical samples collected in this study (see below) were analyzed for virus serotyping and sequencing.

### Patient Consent Statement

Written informed consent was obtained from all patients or their legal guardians prior to blood sample collection. The study protocol was reviewed and approved by the Institutional Review Board of Kaohsiung Medical University (KMUHIRB, approval number: KMUHIRB-E(II)-20240133). All study procedures conformed to the ethical standards of the Declaration of Helsinki and local regulatory requirements.

The analysis of national surveillance data from the Taiwan CDC did not require patient consent, as the data consisted exclusively of de-identified case numbers available in the public domain.

### Case Definitions

Indigenous dengue cases were defined as laboratory-confirmed patients with no international travel history within 14 days prior to illness onset; imported cases were those with a documented travel history to dengue-endemic countries during this period, according to Taiwan CDC classification. Severe dengue (SD) was defined following the 2009 WHO criteria.

### Clinical Sample Collection, Virus Isolation, and Serotyping

From June to December 2023, a total of 1276 suspected dengue patients were enrolled in this study at Kaohsiung Medical University Hospital, and their acute-phase serum samples were collected. Suspected dengue was defined according to the Taiwan CDC/national case definition and mirrored the inclusion criteria used in our previous study [20], namely an acute febrile illness with clinical features compatible with dengue (eg, headache, retro-orbital pain, myalgia/arthralgia, rash, or bleeding tendency) and an epidemiologic link to the ongoing outbreak or travel to a dengue-endemic area. Acute-phase serum was defined as blood drawn during the febrile phase, within 1–7 days after symptom onset and before defervescence. RT-PCR screening identified 206 positive cases. After RT-PCR screening, each positive sample was assigned a computer-generated random number, and 116 samples were then selected by simple random sampling (without stratification) for molecular serotyping and virus isolation.

The details of sample collection, virus isolation, and serotyping were previously described [20, 23, 24].

In brief, patients presenting to Kaohsiung Medical University Hospital with suspected dengue virus infection provided acute-phase blood samples within 1–7 days of symptom onset. Samples were centrifuged at 1500 × *g* for 10 minutes to separate plasma, which was subsequently aliquoted and stored at −80 °C. For viral isolation, plasma was diluted (1:20 to 1:160) in minimum essential medium containing 2% fetal bovine serum, and inoculated onto confluent C6/36 cell monolayers. The cultures were incubated at 28 °C in a 5% CO₂ atmosphere and observed daily for cytopathic effects (CPE) over a 7-day period. If no CPE developed, up to 5 serial blind passages were conducted. The presence of viral replication was confirmed by qRT-PCR detection of viral RNA in the culture supernatants. Serotyping was subsequently performed by NS5-targeted qRT-PCR using primers and probes tailored from established methods [23, 24].

### Phylogenetic Analysis

To determine the genetic characteristics, genotype, and evolutionary context of the 2023 DENV-1 and DENV-2 isolates, we performed phylogenetic analysis based on the complete envelope (E) gene sequence. The protocol was published elsewhere [17]. In brief, viral RNA was extracted from culture supernatants of isolated DENV strains using the QIAamp Viral RNA Mini Kit (Qiagen, Hilden, Germany) following the manufacturer's instructions. The full-length E gene (∼1.5 kb) was amplified using a One-Step RT-PCR Kit (Qiagen, Germany) with previously validated, serotype-specific primer sets [17]. PCR products were purified using a column-based cleanup kit and sequenced bidirectionally using the ABI 3730xl DNA Analyzer (Applied Biosystems). Forward and reverse reads were assembled and manually edited to obtain consensus sequences with Sequencher v5.4.6 (Gene Codes Corporation).

Consensus E-gene sequences of the 2023 Taiwanese isolates were verified and compared using BLASTn against the GenBank database. All reference sequences were retrieved from the NCBI GenBank database and selected according to the following criteria: (1) inclusion of reference strains cited in official Taiwan CDC publications for dengue surveillance and genotype classification; (2) incorporation of previously reported Taiwanese isolates from our earlier studies, representing historical dengue outbreaks in Taiwan; (3) addition of the top BLASTn hits showing the highest sequence similarity to the 2023 isolates, restricted to sequences with complete or near-complete envelope (E) gene coverage; and (4) inclusion of representative epidemic strains from neighboring countries—such as Thailand, Vietnam, the Philippines, and Indonesia—to capture regional genetic diversity and potential transmission linkages.

This curated dataset ensured comprehensive genotypic representation, regional relevance, and robust phylogenetic resolution for comparison with the 2023 outbreak strains.

Multiple sequence alignments were performed using ClustalW implemented in MEGA X software. Phylogenetic trees were constructed using the *neighbor-joining* (*NJ*) method under the *Kimura 2-parameter* nucleotide substitution model, with 1000 bootstrap replicates to assess branch support. The resulting trees were visualized and annotated with FigTree v1.4.4. Detailed information for all sequences, including GenBank accession numbers, origins, years, and genotypes, is provided in Supplementary Tables 1 and 2.

### Whole-genome Sequencing of DENV-1 and DENV-2 Isolates

Viral RNA was extracted from the supernatants of C6/36 cell-infected cultures containing clinical DENV-1 and DENV-2 isolates using the QIAamp Viral RNA Mini Kit (Qiagen, Germany), according to the manufacturer's instructions. For each major outbreak and serotype, 3 independent isolates were selected for full-genome sequencing (2014 DENV-1, 2015 DENV-2, 2023 DENV-1, and 2023 DENV-2; 3 isolates per serotype/year). Full-length genomes were obtained following the protocol described by Christenbury et al [25], which utilizes serotype-specific primers targeting conserved regions of the 3′ untranslated region for cDNA synthesis, followed by PCR amplification of the viral genome in 5 overlapping fragments using a high-fidelity DNA polymerase. The primer sets, PCR conditions, and amplicon sizes were identical to those described by Christenbury et al [25]. Purified PCR products were sequenced by the Sanger method with internal primers to ensure complete coverage. Sequencing chromatograms were examined using BioEdit v7.2.5, and overlapping fragments were assembled into full-length genomes (>10.6 kb) using Vector NTI Advance v11.5.

Among the sequenced isolates, 3 full-length 2023 outbreak-associated DENV-1 genomes (GenBank accession nos. PX460990, PX499695, and PX499696) and 3 full-length 2023 DENV-2 genomes (PX460918, PX499693, and PX499694) were obtained. Likewise, 3 full-length genomes from the 2014 DENV-1 outbreak (accession nos. PX499700, PX499701, and PX499702) and 3 genomes from the 2015 DENV-2 outbreak (accession nos. PX499697, PX499698, and PX499699) were included, encompassing both previously published strains and newly sequenced isolates.

To characterize amino acid mutation patterns across major dengue outbreaks and to assess potential antigenic drift, we generated outbreak- and serotype-specific consensus genomes. For each outbreak–serotype group (2014 DENV-1, 2015 DENV-2, 2023 DENV-1, and 2023 DENV-2), 3 full-length genomes obtained in this study were aligned together with the corresponding Taiwan CDC reference sequence. A consensus sequence was then derived by calling the majority nucleotide at each coding position, thereby reducing sequencing noise and minimizing the impact of rare or potentially artifactual nucleotide changes. The resulting consensus coding sequences were translated into polyprotein amino acid sequences. Although our clinical isolates exhibited minor nucleotide differences relative to the CDC reference genomes, their deduced amino acid sequences were 100% identical within each outbreak–serotype group. Accordingly, each consensus amino acid sequence was used as the representative strain for its respective outbreak, and these 4 consensus polyproteins were subsequently compared with identify nonsynonymous amino acid substitutions between outbreaks.

### Time-lagged Poisson Regression

To evaluate the temporal relationship between potential predictors and the incidence of indigenous dengue cases during the 2023 outbreak, we applied generalized linear models, specifically time-lagged Poisson regression models with a log link function, as previously described [21]. Monthly data on imported and indigenous dengue cases, meteorological variables (average temperature and precipitation), and the BI were collected for Tainan and Kaohsiung from January 2022 to December 2024. The BI, a standard entomological metric for *Aedes* mosquito larval density, is calculated as the number of water-holding containers positive for *Aedes* larvae per 100 inspected houses. Breteau Index data, obtained from Taiwan CDC's routine vector surveillance, were used as a proxy indicator of vector abundance. Monthly population size for each city was included as an offset term in all models to account for population variation. Time lags of 1, 2, and 3 months were applied to each predictor variable to assess delayed effects on dengue incidence. Calendar month and year were included as covariates to control for seasonal and inter-annual variability. Pearson chi-square corrections were used to account for overdispersion. All analyses were conducted using SAS version 9.3 PROC GENMOD (SAS Institute Inc., Cary, NC, USA).

The BI was selected as the entomological predictor for the time-lag regression models because it is the only vector index collected continuously and comprehensively across all administrative districts by the Taiwan CDC. Although the Adult Index (AI) was also available in the national surveillance dataset, the AI data were sparse and temporally discontinuous, with very low values recorded across most weeks—particularly in Tainan. This limited temporal variance and data density rendered the AI unsuitable for inclusion in time-lag analyses. Therefore, the BI was used as the sole entomological predictor, providing the most consistent and representative measure of vector activity for evaluating dengue transmission dynamics in both cities.

We constructed both single-factor (univariate) and multiple-factor (multivariate) Poisson regression models as follows:

- Single-factor model: For each covariate separately, the model was specified as:
  $$\log(E[Y_{t}])=\beta_{0}+\beta_{1}\cdot \mathrm{variabl}e_{t-\mathrm{lag}}+\log\;(\mathrm{Populatio}n_{t})$$

where the variable represents temperature, precipitation, imported cases, or BI. *E*[*Y_t_*] denotes the expected number of cases in month *t,* and lag refers to 1, 2, or 3 months.

- Multiple-factor model (including BI): The model simultaneously included multiple covariates as follows:
  $$\log(E[Y_{t}])=\beta_{0}+\beta_{1}\cdot \mathrm{variabl}e_{t-\mathrm{lag}}+\beta_{2}\cdot BI_{t-\mathrm{lag}}+\log\;(\mathrm{Populatio}n_{t})$$

where variables include temperature, precipitation, and imported cases, and BI represents the BI.

The Akaike information criterion (AIC) was used as a metric to assess and compare models. Lower AIC values indicate a model that provides a better balance between goodness-of-fit and model complexity.

## RESULTS

### Epidemiological Overview of the 2023 Outbreak

During the COVID-19 pandemic (2020–2022), Taiwan implemented stringent border control measures that resulted in only sporadic dengue virus (DENV) cases, primarily classified as imported infections. Following the lifting of these restrictions, beginning 29 September 2022, nationals from visa-exempt countries were permitted to enter Taiwan without prior authorization for nonrestricted activities, including business, academic exchanges, tourism, and family visits. This policy change was followed by a substantial influx of travelers from Southeast Asia.

**Figure 1.**
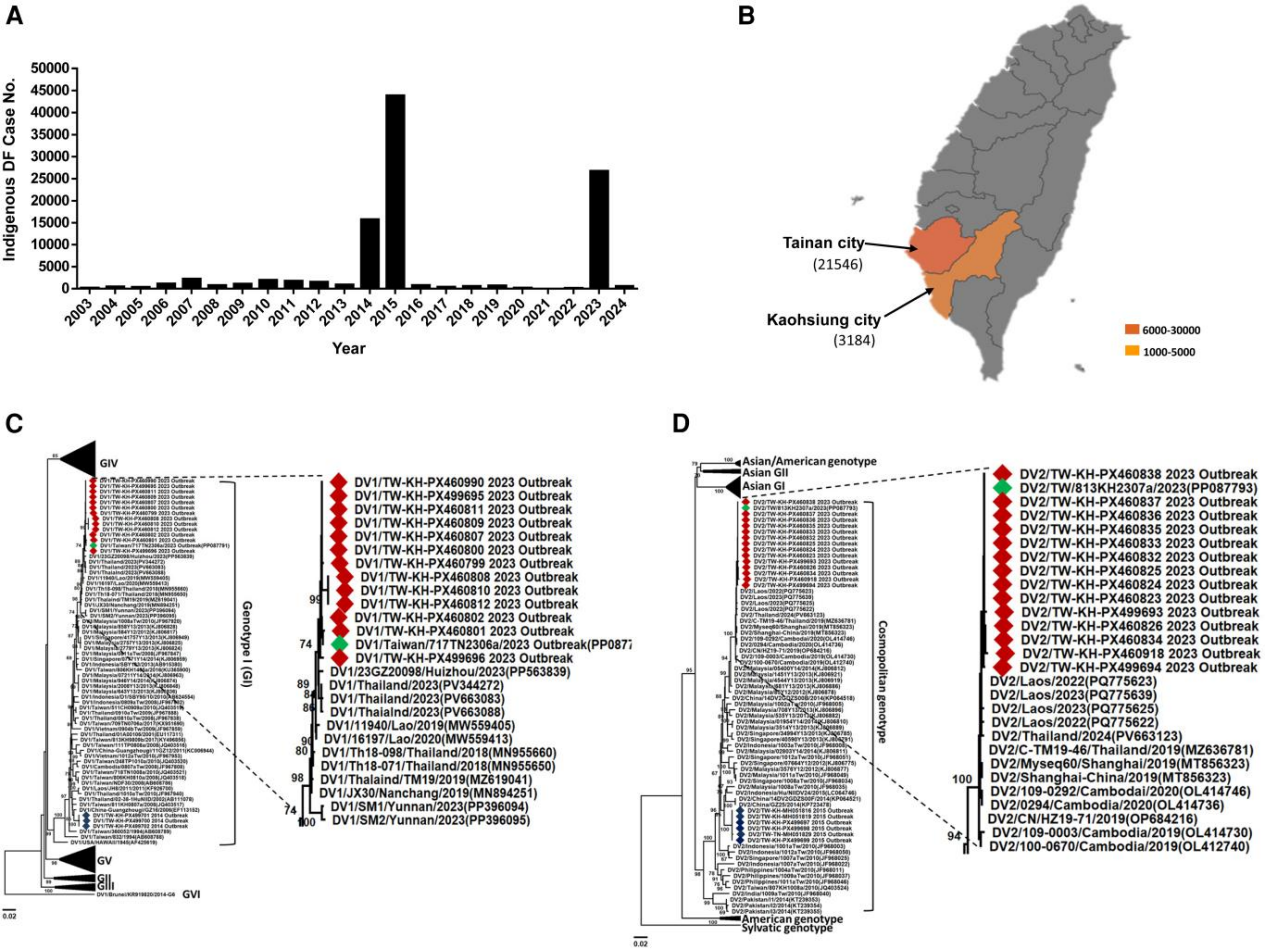
Epidemiological and phylogenetic overview of the 2023 dengue outbreak in Taiwan. *A*, Annual number of laboratory-confirmed indigenous dengue virus (DENV) cases reported in Taiwan from 2003 to 2024. *B*, Geographic distribution of dengue fever cases during the 2023 outbreak, highlighting the 2 most affected southern cities, Tainan and Kaohsiung. *C*, Phylogenetic tree of DENV-1 envelope (E) gene sequences constructed using the neighbor-joining (NJ) method with the Kimura 2-parameter substitution model, showing the genetic relationships of 2023 outbreak-associated isolates. *D*, Phylogenetic tree of DENV-2 envelope (E) gene sequences constructed using the neighbor-joining (NJ) method with the Kimura 2-parameter substitution model, showing the genetic relationships of 2023 outbreak-associated isolates. Red diamonds indicate 2023 clinical isolates obtained in this study, the green diamond represents the 2023 reference isolate reported and registered by the Taiwan Centers for Disease Control (Taiwan CDC), and blue diamonds indicate isolates associated with the 2014 DENV-1 and 2015 DENV-2 outbreaks. GenBank accession numbers of the isolates and reference strains are listed in Supplementary Tables 1 and 2.

A large-scale dengue outbreak subsequently emerged in 2023, resulting in 26 706 laboratory-confirmed cases reported by the Taiwan CDC (Figure 1*A*; Supplementary Table 3). The majority of indigenous cases were concentrated in Tainan City (21 546 cases; 80.7%) and Kaohsiung City (3184 cases; 11.9%) (Figure 1*B*), both of which have historically served as primary epicenters in previous outbreaks. During this period, 137 cases of SD were documented, including 44 deaths, corresponding to a case fatality rate (CFR) of 32.1%. This CFR was markedly higher than those observed in the 2014 (15.4%) and 2015 (24.4%) outbreaks (Supplementary Table 4). A summary of dengue case numbers, SD cases, and fatalities during the 3 major outbreaks in Taiwan over the past decade is presented in Supplementary Table 3. Furthermore, it was noted that following the reopening of borders, multiple travelers infected with dengue from Southeast Asian countries entered Taiwan (Supplementary Fig. 1).

### Serotype Distribution and Phylogenetic Analysis

To better understand the virological characteristics of the outbreak, we enrolled 1276 patients with suspected dengue at Kaohsiung Medical University Hospital and collected acute-phase blood samples, defined as blood drawn during the febrile phase within 1–7 days after symptom onset. Plasma samples were screened for DENV RNA using RT-PCR. Of the 206 RT-PCR–positive samples, each was assigned a computer-generated random number, and 116 were then selected by simple random sampling for molecular serotyping using real-time RT-PCR. Real-time RT-PCR serotyping identified 60 samples (51.7%) as DENV-1 and 56 samples (48.3%) as DENV-2, indicating cocirculation of 2 serotypes during the outbreak (Supplementary Table 5).

Next, to analyze the phylogeny of these clinical isolates, 81 samples (69.8%, 81/116) yielded successful viral isolation in C6/36 mosquito cells. Serotyping of culture-derived isolates showed that 45 (55.6%) were DENV-1 and 36 (44.4%) were DENV-2 (Supplementary Table 5). These isolates were subsequently subjected to RT-PCR to amplify the full-length envelope (E) gene, and the resulting sequences were used for phylogenetic analysis. Phylogenetic trees revealed that outbreak-associated DENV-1 isolates clustered closely with the Taiwan CDC-reported 2023 reference strain (DV1/Taiwan/717TN2306a/2023) and formed a subcluster with isolates from Laos, Thailand, and China (Figure 1*C*). Similarly, DENV-2 isolates grouped with the Taiwan CDC-reported 2023 strain (DV2/TW/813KH2307a/2023) and showed close relationships with strains from Thailand, Cambodia, and China (Figure 1*D*). Regarding the genotype identification, results from phylogenetic trees indicated that the DENV-1 strains from the 2014 and 2023 outbreaks belong to genotype I (GI) (Figure 1*C*), while the DENV-2 strains from the 2015 and 2023 outbreaks belong to the cosmopolitan genotype (Figure 1*D*).

Notably, isolates from imported and indigenous cases were interspersed within the same phylogenetic clusters, indicating that both groups shared high genetic similarity. Overall, sequence similarity and phylogenetic analyses suggested that the 2023 DENV-1 and DENV-2 isolates circulating in Taiwan were genetically related to strains from neighboring Southeast Asian countries, consistent with regional viral diversity.

### Whole-Genome Sequence Comparison of Outbreak Isolates

As noted above, 3 major dengue outbreaks have occurred in Taiwan over the past decade—2014 (DENV-1), 2015 (DENV-2), and 2023 (DENV-1 and DENV-2). Because Taiwan is a dengue-non-endemic region where local transmission ceases each winter and viruses are re-introduced from neighboring endemic countries, these historical epidemic strains provide the most relevant local references for assessing long-term viral divergence. During 2019–2022, dengue activity was minimal (<400 national cases, mostly imported), leaving few intermediate isolates for genomic comparison. To investigate possible antigenic drift and its potential phenotypic implications, we performed full-length genome sequencing and comparative analyses of 2023 isolates against the corresponding epidemic reference strains from 2014 (DENV-1) and 2015 (DENV-2).

**Figure 2.**
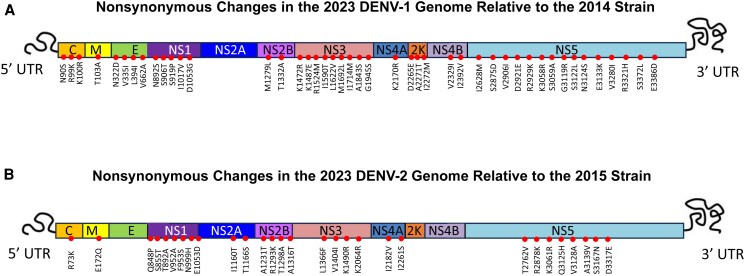
Schematic representation of amino acid substitutions across full-length DENV-1 and DENV-2 polyproteins during the 2023 outbreak in Taiwan. Whole-genome sequences of (*A*) DENV-1 and (*B*) DENV-2 isolates from the 2023 outbreak were compared with 2014 outbreak–associated DENV-1 and 2015 outbreak–associated DENV-2 strains, respectively, and all non-synonymous substitutions in the 2023 viruses are mapped along the complete polyprotein. Amino acid positions and protein domains are drawn to scale according to genome annotation.

A total of 45 nonsynonymous substitutions were identified in 2023 DENV-1 relative to the 2014 reference and 29 in 2023 DENV-2 relative to the 2015 reference (Figure 2 and Table 1).

**Table 1.** Non-Synonymous Nucleotide, Codon, and Amino Acid Differences Across the Full-length Genomes of DENV-1 and DENV-2 Isolates From the 2023 Outbreak Compared With Reference Strains From the 2014 DENV-1 and 2015 DENV-2 Outbreaks, Respectively

DENV Serotype	Nucleotide Position (CDS)	Nucleotide Substitution	2014 Codon	2023 Codon	Codon Change	Protein	Amino Acid Position (Protein-Specific)	Amino Acid Position (Polyprotein)	Amino Acid Mutation (Protein-Specific)	Amino Acid Mutation (polyprotein)
DENV-1	268	A→G	AAC	AGC	AAC→AGC	C	90	90	N90S	N90S
	295	G→A	AGA	AAA	AGA→AAA	C	99	99	R99K	R99K
	298	A→G	AAA	AGA	AAA→AGA	C	100	100	K100R	K100R
	307	A→G	ACC	GCC	ACC→GCC	prM	3	103	T3A	T103A
	994	A→G	AAC	GAC	AAC→GAC	E	42	332	N42D	N332D
	1003	G→A	GTC	ATC	GTC→ATC	E	45	335	V45I	V335I
	1180	C→A	CTA	ATA	CTA→ATA	E	104	394	L104I	L394I
	1984	T→C	GTA	GCA	GTA→GCA	E	372	662	V372A	V662A
	2674	A→G/ T→C	AAT	AGC	AAT→AGC	NS1	98	892	N98S	N892S
	2716	T→A	TCC	ACC	TCC→ACC	NS1	112	906	S112T	S906T
	2755	T→C	TCT	CCT	TCT→CCT	NS1	125	919	S125P	S919P
	3049	A→G	ATA	GTA	ATA→GTA	NS1	223	1017	I223V	I1017V
	3157	A→G	GAT	GGT	GAT→GGT	NS1	259	1053	D259G	D1053G
	3835	A→T	ATG	TTG	ATG→TTG	NS2B	49	1279	M49L	M1279L
	3994	A→G	ACC	GCC	ACC→GCC	NS2B	102	1332	T102A	T1332A
	4414	A→G	AAG	AGG	AAG→AGG	NS3	123	1472	K123R	K1472R
	4459	A→G	AAA	GAA	AAA→GAA	NS3	138	1487	K138E	K1487E
	4570	G→T	AGG	ATG	AGG→ATG	NS3	175	1524	R175M	R1524M
	4768	T→C	ATC	ACC	ATC→ACC	NS3	241	1590	I241T	I1590T
	4864	T→G	TTA	GTA	TTA→GTA	NS3	273	1622	L273V	L1622V
	5074	A→C	ATG	CTG	ATG→CTG	NS3	343	1692	M343L	M1692L
	5140	A→G	ATA	ATG	ATA→ATG	NS3	365	1714	I365M	I1714M
	5527	G→T	GCA	TCA	GCA→TCA	NS3	494	1843	A494S	A1843S
	5833	G→A	GGT	AGT	GGT→AGT	NS3	596	1945	G596S	G1945S
	6508	A→G	AAG	AGG	AAG→AGG	NS4A	101	2170	K101R	K2170R
	6793	T→A	GAT	GAA	GAT→GAA	2K	3	2265	D3E	D2265E
	6811	G→A	GCA	ACA	GCA→ACA	2K	9	2271	A9T	A2271T
	6814	A→G	ATA	ATG	ATA→ATG	2K	10	2272	I10M	I2272M
	6985	G→A	GTA	ATA	GTA→ATA	NS4B	47	2329	V47I	V2329I
	7174	A→G	ATT	GTT	ATT→GTT	NS4B	110	2392	I110V	I2392V
	7882	A→G	ATA	ATG	ATA→ATG	NS5	108	2628	I108M	I2628M
	8623	A→G/ G→A	AGT	GAT	AGT→GAT	NS5	355	2875	S355D	S2875D
	8716	G→A	GTT	ATT	GTT→ATT	NS5	386	2906	V386I	V2906I
	8761	C→G	GAC	GAG	GAC→GAG	NS5	401	2921	D401E	D2921E
	8785	G→A	AGA	AAA	AGA→AAA	NS5	409	2929	R409K	R2929K
	9172	A→G	AAG	AGG	AAG→AGG	NS5	538	3058	K538R	K3058R
	9175	T→G	TCA	GCA	TCA→GCA	NS5	539	3059	S539A	S3059A
	9355	G→A	GGA	AGA	GGA→AGA	NS5	599	3119	G599R	G3119R
	9364	C→T	TCA	TTA	TCA→TTA	NS5	602	3122	S602L	S3122L
	9370	A→G/ C→T	AAC	AGT	AAC→AGT	NS5	604	3124	N604S	N3124S
	9397	G→A	GAG	AAG	GAG→AAG	NS5	613	3133	E613K	E3133K
	9838	G→A/ T→C	GTT	ATC	GTT→ATC	NS5	760	3280	V760I	V3280I
	9961	G→A	CGT	CAT	CGT→CAT	NS5	801	3321	R801H	R3321H
	10 114	T→C/ C→T/ G→A	TCG	CTA	TCG→CTA	NS5	871	3372	S852L	S3372L
	10 156	A→T	GAA	GAT	GAA→GAT	NS5	885	3386	E885D	E3386D
**DENV-2**	217	G→A	AGA	AAA	AGA→AAA	C	73	73	R73K	R73K
	514	G→C	GAG	CAG	GAG→CAG	prM	72	172	E72Q	E172Q
	2542	A→C	CAA	CCA	CAA→CCA	NS1	54	848	Q54P	Q848P
	2563	T→A	TCA	ACA	TCA→ACA	NS1	61	855	S61T	S855T
	2674	A→G	ACA	GCA	ACA→GCA	NS1	98	892	T98A	T892A
	2854	T→C	GTA	GCA	GTA→GCA	NS1	158	952	V158A	V952A
	2857	T→C	TTT	TCT	TTT→TCT	NS1	159	953	F159S	F953S
	2995	A→C	AAC	CAC	AAC→CAC	NS1	205	999	N205H	N999H
	3157	A→T	GAA	GAT	GAA→GAT	NS1	259	1053	E259D	E1053D
	3478	T→C	ATA	ACA	ATA→ACA	NS2A	36	1160	I36T	I1160T
	3496	A→T	ACT	TCT	ACT→TCT	NS2A	42	1166	T42S	T1166S
	3691	G→A	GCC	ACC	GCC→ACC	NS2B	1	1231	A1T	A1231T
	3877	G→A	AGA	AAA	AGA→AAA	NS2B	63	1293	R63K	R1293K
	3892	A→G	ACA	GCA	ACA→GCA	NS2B	68	1298	T68A	T1298A
	3946	G→A	GCG	ACG	GCG→ACG	NS2B	86	1316	A86T	A1316T
	4096	C→T	CTC	TTC	CTC→TTC	NS3	17	1366	L17F	L1366F
	4210	G→A	GTA	ATA	GTA→ATA	NS3	55	1404	V55I	V1404I
	4468	A→G	AAG	AGG	AAG→AGG	NS3	141	1490	K141R	K1490R
	6190	A→G	AAG	AGG	AAG→AGG	NS3	715	2064	K715R	K2064R
	6544	A→G	ATC	GTC	ATC→GTC	NS4A	113	2182	I113V	I2182V
	6781	T→G/ T→C	ATT	AGC	ATT→AGC	NS4A	192	2261	I192S	I2261S
	8284	A→G/ C→T	ACA	GTA	ACA→GTA	NS5	242	2762	T242V	T2762V
	8632	G→A	AGG	AAG	AGG→AAG	NS5	358	2878	R358K	R2878K
	9181	A→G	AAA	AGA	AAA→AGA	NS5	541	3061	K541R	K3061R
	9373	A→C	CAA	CAC	CAA→CAC	NS5	605	3125	Q605H	Q3125H
	9382	T→C	GTC	GCC	GTC→GCC	NS5	608	3128	V608A	V3128A
	9415	C→T	GCA	GTA	GCA→GTA	NS5	619	3139	A619V	A3139V
	9499	G→A	AGC	AAC	AGC→AAC	NS5	647	3167	S647N	S3167N
	9949	C→A	GAC	GAA	GAC→GAA	NS5	797	3317	D797E	D3317E

Nucleotide positions are numbered according to the coding sequence (CDS) of the full-length viral genome. Protein-specific amino acid positions and mutations are numbered within each individual viral protein, whereas polyprotein positions and mutations are numbered according to the full-length polyprotein of the reference DENV-1 and DENV-2 strains. Only nonsynonymous mutations are listed. DENV-1 past outbreak isolates correspond to 2014 epidemic strains, and DENV-2 past outbreak isolates correspond to 2015 epidemic strains.

For DENV-1, substitutions were distributed across structural (C, prM, E) and nonstructural (NS1, NS2B, NS3, NS4A, 2K, NS4B, and NS5) proteins. Key changes included multiple substitutions in the envelope protein (N42D, V45I, L104I, and V372A) and NS1 (N98S, S112T, S125P, I223V, D259G). Both NS3 and NS5 accumulated a high number of replacements. Notably, NS5 showed a cluster of substitutions (eg, I108M, S355D, V386I, D401E, R409K, G599R, S602L, N604S, V760I) located within domains involved in RNA-dependent RNA polymerase (RdRp) function, suggesting potential impacts on viral fitness (Figure 2 and Table 1).

Similarly, 2023 DENV-2 isolates exhibited amino acid changes spanning C, prM, NS1, NS2A, NS2B, NS3, NS4A, and NS5. Notable substitutions were clustered in NS1 (Q54P, S61T, T98A, V158A, F159S, N205H, E259D). Other changes were identified in prM (E72Q), NS2A (I36T, T42S), NS2B (A1T, R63K, T68A, A86T), NS3 (L17F, V55I, K141R, K715R), and NS5 (T242V, R358K, K541R, Q605H, V608A, A619V, S647N, D797E). Many of these NS3 and NS5 changes localize to helicase and polymerase regions critical for enzymatic activity and immune modulation in flaviviruses, raising the possibility of altered replication kinetics or host interactions (Figure 2 and Table 1).

Because clinical outcome in dengue is strongly modulated by prior infection history and host immunity, direct genotype-to-phenotype correlation is difficult. Instead, we compared outbreak-level epidemiologic indicators—severe dengue (SD) and mortality rates—across the 3 major outbreaks (see Supplementary Table 4, “Three Dengue Outbreaks in Taiwan Over the Past Decade”). National surveillance data show that although the 2015 DENV-2 epidemic had the largest case count, the 2023 outbreak (DENV-1 and DENV-2 cocirculation) exhibited a relatively higher proportion of severe and fatal cases, suggesting continued viral and host-immunity interplay influencing epidemic severity.

### Imported Cases and Potential Sources of Virus Introduction

Epidemiological surveillance of DENV-positive travelers from late September 2022 to May 2023 identified Vietnam, Indonesia, Malaysia, Thailand, and the Philippines as the major countries of origin for imported cases (Supplementary Fig. 1). National surveillance data from January to December 2023 revealed that the majority of imported cases were associated with travel to Indonesia, Malaysia, Thailand, Vietnam, and India (Supplementary Fig. 2A).

A closer examination of the preoutbreak period (January–May 2023) indicated that most imported infections originated from travelers returning from Indonesia, Malaysia, Thailand, and Vietnam (Supplementary Fig. 2B). These countries accounted for the largest proportion of imported cases during this period, while fewer cases were associated with travel to India and the Philippines.

### Time-Lagged Regression Analysis of Transmission Drivers

**Figure 3.**
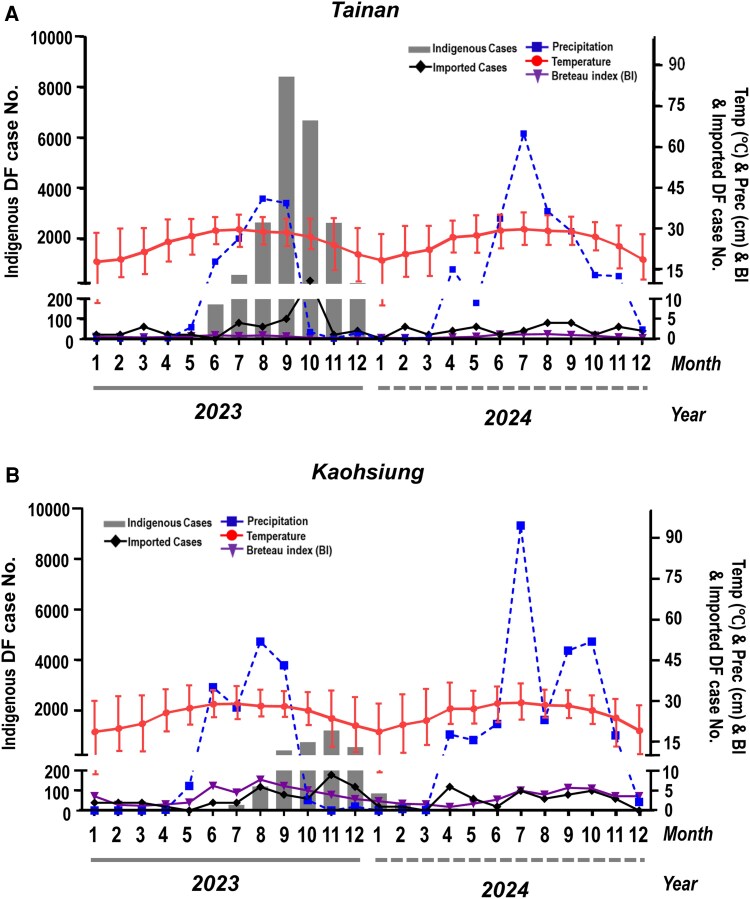
Climatic variation, mosquito density, and imported cases associated with the 2023 dengue outbreak in 2 major southern cities of Taiwan. *A*, Monthly reported dengue cases in Tainan during 2023–2024, shown together with ambient temperature, precipitation, and the Breteau Index. Critically, the last indigenous cases of the main epidemic wave were detected in February 2024; following this date, the absence of indigenous case bars represents a true value of zero reported cases. *B*, Monthly reported dengue cases in Kaohsiung during 2023–2024, shown together with ambient temperature, precipitation, and the Breteau Index. The primary outbreak ceased in April 2024, though sporadic cases were reported subsequently in June (2 cases), November (2 cases), and December (4 cases). Abbreviations: DF, dengue fever; Temp, temperature; Prec, precipitation; BI, Breteau Index.

To assess the drivers of dengue transmission during the 2023 outbreak, we evaluated the associations between indigenous case incidence and potential predictors, including ambient temperature, precipitation, imported dengue cases, and the BI. Analyses were performed separately for Tainan and Kaohsiung using time-lagged models with 1–3-month lag intervals. All models showed a substantially better fit (lower AIC) than the null models (Table 2). Monthly patterns of imported cases, temperature, precipitation, and BI for Tainan and Kaohsiung during the 2023 major outbreak are shown in Figure 3, and those for past large outbreaks are shown in Supplementary Figure 3, highlighting marked seasonal and inter-city variation in these established dengue risk factors.

**Table 2.** Time-Lag Analysis of Factors Associated With Indigenous Dengue Incidence During the 2023 Outbreak in Tainan and Kaohsiung

City	Model	Time Lag	Temperature		Precipitation		Imported Case		Breteau Index (BI)	
			*β*	*P* Value	*β*	*P* Value	*β*	*P* Value	*β*	*P* Value
Kaohsiung	Single factor	lag 1 m	.549	.5685	.002	.0668	.063	<.0001	.190	.4389
		lag 2 m	.122	.9014	.001	.3155	.045	.0002	.812	<.0001
		lag 3 m	.544	.4432	.006	<.0001	.037	<.0001	.363	.0341
	AIC		489.59		406.42		341.19		295.70	
	Multiple factors with BI	lag 1 m	−.103	.6164	−.002	.5217	.014	.7285	…	…
		lag 2 m	.559	.0140	−.001	.9003	−.049	.3046	…	…
		lag 3 m	−1.100	.3720	.001	.7961	.009	.6842	…	…
		BI lag 1 m	.815	.3599	.473	.6339	−.054	.8816	…	…
		BI lag 2 m	.598	.0004	.418	.0279	.331	.5024	…	…
		BI lag 3 m	.679	.0896	.2550	.7848	.973	.0387	…	…
	AIC	…	110.03		109.31		165.23		…	…
Tainan	Single factor	lag 1 m	.228	.2521	.003	.0004	.098	.0036	1.929	.0296
		lag 2 m	.224	.2549	.007	<.0001	.038	.0741	6.108	<.0001
		lag 3 m	.559	<.0001	.003	.0422	−.010	.6151	5.634	<.0001
	AIC	…	685.24		1103.10		3937.67		825.94	
	Multiple factors with BI	lag 1 m	−.396	.5380	.006	<.0001	−.070	.2124	…	…
		lag 2 m	1.879	.2342	−.022	<.0001	.052	<.001	…	…
		lag 3 m	−.110	.8232	−.010	.0001	.023	.5026	…	…
	…	BI lag 1 m	−3.862	.0168	−1.580	.0070	.634	.7709	…	…
	…	BI lag 2 m	−4.707	.3152	12.018	<.0001	3.643	.3760	…	…
	…	BI lag 3 m	−4.607	.3883	29.084	<.0001	9.761	.0134	…	…
	AIC	…	206.54		91.27		138.73		…	…

Beta (*β*) takes e to estimate incidence rate ratio. “m” indicates the month.

The AIC for the null model is 44 560.31 for Tainan and 5736.40 for Kaohsiung.

Abbreviation: AIC, Akaike information criterion (smaller is better).

In Kaohsiung, the single-factor analysis showed that imported cases were significantly associated with indigenous incidence across all lag intervals (lag 1m: *β* = .063, *P* < .0001; lag 2m: *β* = .045, *P* = .0002; lag 3m: *β* = .037, *P* < .0001). The BI exhibited strong positive associations at 2- and 3-month lags (lag 2m: *β* = .812, *P* < .0001; lag 3m: *β* = .363, *P* = .0341). Precipitation was significant only at the 3-month lag (*β* = .006, *P* < .0001), while temperature showed no significant associations. The BI model set yielded the lowest AIC (295.70) among single-predictor models, indicating the best fit. In the multiple-factor analysis, the model incorporating BI with a 2-month lag was the best-fitting model (AIC = 109.31). This indicates that the entomological situation, as captured by BI, is a critical component for building the best predictive model in Kaohsiung. Within this optimal model, both temperature (*β* = .598, *P* = .0004) and precipitation (*β* = .418, *P* = .0279) were identified as significant independent predictors of dengue incidence.

In Tainan, the single-factor models showed that precipitation and the BI were significant positive predictors at all 3-time lags. The BI showed particularly strong associations at 2- and 3-month lags (lag 2m: *β* = 6.108, *P* < .0001; lag 3m: *β* = 5.634, *P* < .0001). Temperature was significant at the 3-month lag (*β* = .559, *P* < .0001), and imported cases were significant at the 1-month lag (*β* = .098, *P* = .0036). However, the multivariate analysis revealed a different dynamic compared with Kaohsiung. While BI was a highly significant positive predictor in single-factor analyses, in the best-fitting multiple-factor model (including BI with a 2-month lag, AIC = 91.27), precipitation emerged as the single most dominant and highly significant independent predictor (*β* = 12.018, *P* < .0001). These results indicate that in Tainan, the explanatory power of the BI is subsumed by precipitation, reflecting the strong correlation between rainfall and transmission in this region. This aligns with the epidemiological observation that Tainan's largest outbreaks were preceded by extreme precipitation events. Precipitation was also the most significant factor in the 3-month lag model (*β* = 29.084, *P* < .0001), which also showed a significant effect from imported cases (*β* = 9.761, *P* = .0134) (Table 2).

## DISCUSSION

Taiwan, situated at the intersection of East and Southeast Asia, serves as a critical hub for international travel and commerce, making it particularly vulnerable to the introduction of communicable diseases such as dengue [26]. Although *Aedes* mosquitoes—the primary vector for dengue virus (DENV)—are well established in Taiwan, dengue is still classified as a non-endemic disease by the Taiwan CDC [9]. In most years, fewer than 400 indigenous dengue cases are reported (Figure 1 and Supplementary Table 1), with limited or no evidence of sustained local transmission.

During 2020–2022, dengue activity in Taiwan was extremely low, coinciding with the period of stringent COVID-19 border control and non-pharmaceutical interventions. These measures almost certainly reduced the importation and local transmission of dengue viruses; however, they may also have affected dengue surveillance. Changes in health-care–seeking behavior, temporary service disruptions, and the prioritization of COVID-19 testing could have contributed to under-ascertainment of dengue during 2020–2021. Thus, the apparent trough in dengue incidence between the 2015 and 2023 outbreaks likely reflects a combination of genuinely reduced transmission and, to an uncertain extent, reduced case detection.

Notably, the proportion of SD cases and related deaths was higher in 2023 than in previous outbreaks. Among 137 SD cases, 44 deaths were reported, yielding an SD CFR of 32.1%, which exceeded those of the 2014 (15.4%) and 2015 (24.4%) outbreaks (Supplementary Table 2). Although the absolute number of SD cases was smaller in 2023, this disproportionately high fatality suggests that infections increasingly occurred in older and medically fragile populations. Nationwide analyses of dengue in Taiwan from 2003 to 2013 and the 2014–2015 dengue outbreaks have consistently shown that age ≥60 years and comorbidities are key determinants of both progression to DHF (or SD) and death, with DM emerging as an independent predictor of mortality among DHF cases [12, 27–29].

During the more severe 2015 dengue outbreak in southern Taiwan, older age, diabetes, and hypertension were again identified as major clinical risk factors for SD and ICU admission [20, 30, 31]. Large population-based and NHIRD-based studies have further confirmed that DM and other chronic cardiometabolic conditions significantly increase the risks of SD, prolonged hospitalization, and death in Taiwanese patients [32]. Emerging data from the 2023 outbreak point in the same direction. A recent cohort study from Kaohsiung identified older age, secondary infection (pre-existing anti-DENV IgG), and chronic diseases—including DM and renal disease—as major risk factors for SD and mortality in 2023 [33]. These findings are consistent with earlier seroepidemiological work showing high baseline DENV seroprevalence in adults in Kaohsiung, implying that many older patients infected in 2023 were experiencing secondary infections on top of multiple comorbidities [34].

Previous studies demonstrated that secondary dengue infection in Taiwan markedly increases the risk of severe disease, particularly when the interval between infections exceeds 2 years, providing epidemiologic support for an ADE-like mechanism in an aging, previously exposed population [31, 35]. Together with our observation of cocirculating DENV-1 and DENV-2 in 2023, this suggests that ADE driven by heterotypic secondary infection likely amplified the impact of traditional host risk factors (advanced age, DM, hypertension), contributing to the high SD case fatality rate. In addition, obesity has been increasingly recognized as a risk factor or modifier of dengue severity: meta-analyses have shown approximately 1.5-fold higher odds of SD among obese patients, and both WHO guidance and Taiwanese clinical reviews list obesity as a condition warranting closer monitoring because of more pronounced thrombocytopenia, hemoconcentration, and hepatic injury [36]. Although BMI data were not systematically available in our dataset, the growing prevalence of obesity and metabolic syndrome in Taiwan likely further interacts with aging, DM, hypertension, and prior DENV exposure to shape the vulnerability profile of patients infected during the 2023 outbreak.

It is also noteworthy that a distinguishing feature of the 2023 outbreak was the cocirculation of DENV-1 and DENV-2, a phenomenon rarely observed in Taiwan, which may be associated with the high number of SD cases and increased mortality (Supplementary Table 1). Cocirculation of multiple serotypes increases the risk of severe disease due to antibody-dependent enhancement (ADE), where pre-existing, non-neutralizing antibodies from a prior infection enhance viral entry and replication during secondary infection [37, 38]. Past studies have linked serotype cocirculation to increased disease severity and hospitalization. For example, a 2008–2009 outbreak in Vietnam involving DENV-1, DENV-2, and DENV-4 was associated with higher rates of SD [39, 40]. Coinfections involving 2 or even 3 dengue virus serotypes in humans have also been reported in Puerto Rico [41] and Sri Lanka [42]. Although rare, recombination between serotypes has been documented and may impact viral evolution, virulence, or immune escape [43, 44]. These findings highlight the importance of serotype surveillance and genomic monitoring to better anticipate outbreak dynamics and disease severity.

In our cohort, only 16.1% of clinically suspected dengue cases were RT-PCR–positive. This modest positivity rate should be interpreted in the context of our surveillance setting rather than as a limitation of the assay itself. First, patients were enrolled according to a broad “suspected dengue” definition used in routine surveillance, which is designed to be highly sensitive and therefore includes a substantial proportion of non-dengue febrile illnesses. Second, the performance of RT-PCR is strongly dependent on the day of illness at sampling; although we aimed to collect acute-phase specimens within 1–7 days after symptom onset, heterogeneity in presentation time means that some samples were likely obtained when viremia had already begun to decline, reducing detection sensitivity. In addition, differences in viremia dynamics between primary and secondary infections, as well as preanalytical factors such as sample type (blood, serum, or plasma), storage, and transport conditions, may further contribute to false-negative RT-PCR results in true dengue cases. Overall, the RT-PCR positivity observed in our study is comparable to that reported in other hospital-based surveillance cohorts, where only a minority ( approximately 15%–30%) of clinically suspected dengue cases test positive by RT-PCR, and is consistent with known determinants of assay yield such as timing of sampling, viremia level, and preanalytical factors [45–47].

Overall, the RT-PCR positivity observed in our study is comparable to that reported in similar hospital-based surveillance cohorts [33] and is consistent with these known determinants of assay yield.

Our full-genome comparison of the 2023 DENV-1 and DENV-2 outbreak isolates with isolates from previous epidemics (2014 for DENV-1 and 2015 for DENV-2) revealed several nonsynonymous amino acid substitutions with potential functional relevance (Figure 2 & Table 1). In DENV-1, mutations such as D132N—located within the fusion loop region of the envelope protein—have been reported to alter antibody binding and facilitate immune escape, potentially contributing to increased viral fitness [48–50]. Similarly, the G188D substitution in NS2A and the S75P substitution in NS2B are noteworthy, as NS2A is involved in RNA replication and virion assembly, while NS2B serves as an essential cofactor for NS3 protease activity; both alterations may impact replication efficiency and immune evasion [51, 52]. Mutations within NS3 (R269K) and NS5 (R943K, R1255K) occur near functional motifs involved in helicase activity, RNA synthesis, and interferon antagonism, which are key determinants of viral replication and host immune suppression [52, 53]. In DENV-2, we identified substitutions in structural and nonstructural proteins, including prM-Q172E, NS1-P848Q/A892T, NS3-T1231A/K1293R/T1316A, and multiple changes in NS5 (T2260A, K2322R, F2377L, R2498K). NS1 mutations may affect protein secretion and complement antagonism, while NS3 and NS5 alterations may modulate enzymatic functions, replication kinetics, and interferon antagonism based on analogous studies in related flaviviruses [52, 54, 55]. Collectively, these amino acid changes may influence viral pathogenicity, transmission dynamics, or antigenic properties. While the functional consequences of these mutations require experimental validation, their presence in outbreak isolates highlights potential adaptive changes associated with viral persistence and epidemic spread.

A comparative analysis of the 2023 outbreak (Figure 3) against the 2014–2015 epidemics (Supplementary Fig 3) reveals significant regional heterogeneity and highlights the potential impact of extreme climatic events on outbreak magnitude. The figures clearly show that the primary epidemic focus shifts between cities; the 2014 outbreak was predominantly a Kaohsiung-centric event, whereas the 2015 and 2023 outbreaks were both disproportionately centered in Tainan. This observed pattern of shifting epicenters strongly validates our study's approach of performing city-specific regression analyses, demonstrating that monolithic, Taiwan-wide models would fail to capture these critical, local transmission dynamics.

Furthermore, this comparison suggests a potential association between extreme climatic events and outbreak *magnitude*. In Tainan, the 2 largest epidemics on record (2015 and 2023) were both immediately preceded by an anomalous, extreme spike in Precipitation (Prec) in August. This finding suggests that while baseline seasonal conditions—such as high Temperature (Temp) and an elevated BI—create the necessary “fuel” for transmission, such extreme rainfall events may act as an epidemic multiplier. They likely lead to an explosive increase in vector breeding sites and, consequently, a far larger and more rapid outbreak scale.

Following the large 2014–2015 epidemics, Taiwan implemented strengthened national and municipal dengue control programs, including intensified source-reduction campaigns, community cleanup activities, enhanced risk communication, and more systematic vector surveillance. These public health interventions likely contributed to the relatively low dengue activity observed in the intervening years and may also have influenced entomological indicators such as the BI by reducing larval habitats and stabilizing vector densities. Although our study was not designed to formally evaluate the impact of specific control measures, the temporal trends in BI and case counts across 2014–2016 and 2023–2024 are compatible with an ongoing interaction between control efforts, climatic variability, and viral importation in shaping dengue risk. Future work that integrates more detailed intervention data will be important to disentangle these effects and to quantify the impact of individual control components.

In addition, our results revealed key insights into the multifactorial drivers of indigenous DENV transmission (Figure 3 and Table 2). A critical finding, shown in Table 2, is that imported cases were highly significant predictors in single-factor models, but this significance disappeared in the multiple factors models. This does not diminish their importance; rather, it clarifies their role as the “spark” or *trigger* for outbreaks. The environmental factors—BI, Temperature, and Precipitation—act as the “fuel.” The multivariate model shows that the *scale* of an outbreak is best predicted by the amount of “fuel,” and the statistical signal of the “spark” is overshadowed once the “fuel” is accounted for.

This interpretation also addresses 2 other possibilities. First, as a limitation, the under-reporting of asymptomatic or subclinical missed imported cases would inherently weaken this variable's statistical signal. Second, this “spark and fuel” model refutes the hypothesis of a hidden endemic cycle. An endemic cycle would require year-round transmission, yet our data in Figure 3 clearly shows a complete seasonal cessation of cases in 2024. This, combined with our phylogenetic data showing newly introduced strains, confirms that imported cases are the necessary *stochastic triggers* for seasonal epidemics in a non-endemic setting

Although BI was used as the primary entomological predictor in the current study, adult indices (AI) are conceptually more closely correlated with the true biting mosquito population and thus better reflect the “real” vector situation in the field. However, in our dataset, adult mosquito surveillance variables (such as AI) exhibited extremely low counts and limited temporal coverage, consistent with the nationwide reporting pattern described in the Methods. By contrast, the BI is the only entomological metric collected continuously and comprehensively across all administrative districts by the Taiwan CDC. This consistency makes BI the most robust and policy-relevant entomological indicator currently available for long-term modeling. Despite its known limitations as a larval index, previous studies and our current data support its epidemiological validity within Taiwan's surveillance system. In our analyses, BI and AI exhibited moderate to strong temporal correlations (r = 0.73 in Kaohsiung; r = 0.58 in Tainan; both *P* < .001), indicating that larval density trends reliably mirror adult vector abundance. Therefore, BI serves as a practical and representative entomological surrogate for evaluating dengue transmission risk in city-level time-lagged analyses.

This study further revealed complex, lag-dependent roles of environmental variables. For example, precipitation showed variable effects depending on the lag and model structure—being positively associated with incidence in several single-predictor models, but reversing to negative associations in certain multivariable models (Table 2). This pattern likely reflects dual ecological mechanisms: moderate rainfall supports breeding site formation, whereas excessive precipitation can flush out larvae and temporarily suppress vector populations. Similarly, temperature—known to influence viral replication and vector development—exhibited inconsistent associations across cities and model specifications, suggesting nonlinear effects and interactions with other factors such as humidity, water storage behaviors, or human mobility. Taken together, these findings underscore the importance of localized, context-specific approaches to dengue surveillance and control. The contrasting roles of BI and precipitation between Kaohsiung and Tainan highlight how DENV transmission dynamics are shaped by distinct environmental, ecological, and possibly socio-demographic conditions, and argue against a 1-size-fits-all reliance on any single indicator.

This study has several limitations that should be acknowledged. First, our genomic analyses were based on a relatively small number of complete genomes per outbreak–serotype and on consensus sequences derived from these isolates. Although phylogenetic analysis confirmed that these genomes clustered with the official Taiwan CDC reference strains and are therefore representative of the dominant genotypes, this sampling strategy cannot fully capture the intra-outbreak viral diversity or rare adaptive mutations that may have circulated during the 2023 epidemic. In addition, both the molecular and regression analyses were restricted to 2 southern cities (Tainan and Kaohsiung); as such, our findings should be interpreted as city-specific and may not be directly generalizable to other regions of Taiwan or to different transmission settings.

Second, while our genomic analysis identified several nonsynonymous mutations in the 2023 isolates, our discussion of their potential impact on viral replication or immune evasion is speculative. The actual functional consequences of these specific amino acid substitutions were not experimentally validated. Further in vitro functional assays and in vivo virulence studies are necessary to confirm the precise effect of these mutations on viral fitness and pathogenicity.

Finally, our time-lagged regression models used the BI as the sole entomological predictor. Although adult mosquito indices (like the AI) were available, our analysis (as shown in Supplementary Figures R1 and R2) found this data to be exceptionally sparse and lacking in temporal variance, particularly in Tainan. The BI was the only vector index collected consistently and comprehensively enough to be suitable for the time-lag analysis. While our data showed a moderate correlation between BI and AI, future models could be improved by integrating more robust adult vector surveillance data if it becomes available.

## CONCLUSIONS

The resurgence of dengue in Taiwan in 2023, after the COVID-19 pandemic, underscores the transboundary nature of vector-borne diseases in the post-pandemic era. Our findings highlight the critical roles of imported cases, cocirculating DENV-1 and DENV-2 strains phylogenetically linked to Southeast Asia, favorable climatic conditions, and increased mosquito density in facilitating local transmission. Time-lagged models revealed marked regional heterogeneity in predictive factors, with vector indices playing a dominant role in Kaohsiung, whereas meteorological variables exerted stronger influence in Tainan. These results emphasize the need for region-specific vector surveillance, integrated climate-informed early warning systems, and cross-border collaboration to prevent future outbreaks. Strengthening international dengue monitoring and ensuring timely genomic characterization of circulating strains will be essential to mitigate the global impact of dengue resurgence.

## Supplementary Material

ofag070_Supplementary_Data

## Supplementary Data

Supplementary materials are available at *Open Forum Infectious Diseases* online. Consisting of data provided by the authors to benefit the reader, the posted materials are not copyedited and are the sole responsibility of the authors, so questions or comments should be addressed to the corresponding author.

## Notes

***Author Contributions*.** S. -F. Wa. conceived and designed the study. J. -Y. H. and Z. -S. Y. conducted the data analysis. S. -F. We. performed the statistical analysis verification. J. -Y. H., S. -F. We., Y. -W. T., and Z. -S. Y. prepared the original draft and performed subsequent revisions. W. A., A. T., D. -Y. C., W. -H. W., Y. -H. C., and C. -Y. L. extensively reviewed and edited the manuscript. All authors have read and agreed to the published version of the manuscript.

***Acknowledgments.*** The authors thank the Center for Laboratory Animals in Kaohsiung Medical University for the animal care and the Center for Research Resources and Development in Kaohsiung Medical University for the assistance in protein identification, flow cytometry, and confocal image analysis.

***Financial support.*** This work was supported by grants from the National Science and Technology Council, R.O.C. (NSTC 112-2320-B-037-032-MY3, NSTC 113-2740-M-037-001, and NSTC 112-2740-M-037-001 to S. -F. W.; NSTC 112-2628-B-037-003 to C. -Y. L.), the Kaohsiung Medical University Research Center (KMU-TC114B01), the Kaohsiung Medical University Research Foundation (KMU-M112007), the NYCU-KMU Joint Research Project (NYCU-KMU-113-I004), and the NSYSU-KMU Joint Research Project (#NSYSUKMU 113-I04).

***Data availability*.** Publicly available datasets were analyzed in this study. The epidemiological and vector surveillance data can be found in the Taiwan Centers for Disease Control Open Data Platform (https://www.cdc.gov.tw). Meteorological data are available from the Central Weather Administration (https://www.cwa.gov.tw). The viral genome sequences generated in this study have been deposited in GenBank under accession numbers PX460990–PX460992 (DENV-1) and PX460918, PX499693–PX499694 (DENV-2). All other relevant data are within the manuscript and its Supplementary Information files.
