# Supplementary material for: A Large Dengue Outbreak in Taiwan, 2023: Driven by Imported Cases, Serotype Cocirculation, and Climate Variability
Source: Open Forum Infect Dis. 2026 Feb 21;13(3):ofag070. doi: 10.1093/ofid/ofag070 (PMC12964117; doi:10.1093/ofid/ofag070)
Supplement: ofag070_Supplementary_Data [file ofag070_supplementary_data.docx]

**Supplementary data**

**
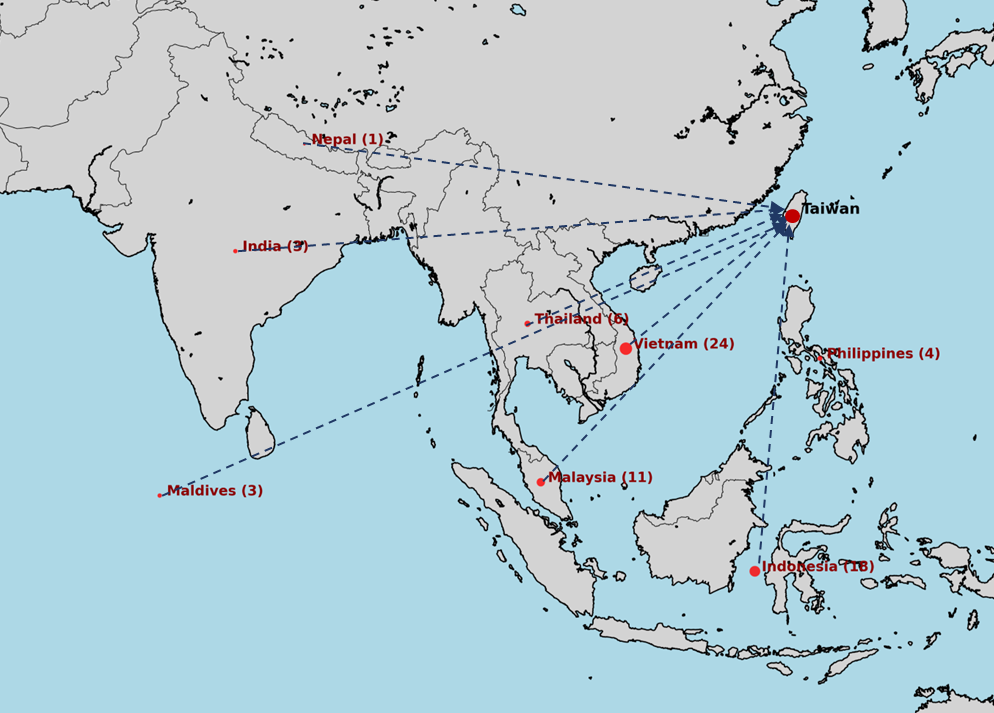
**

**S-Figure 1. Imported dengue cases from neighboring countries to Taiwan in the post–COVID-19 period.**

Countries identified as major sources of imported dengue cases following the reopening of Taiwan’s borders after COVID-19 are shown. Data from the Taiwan CDC indicate 525 imported dengue cases recorded between late September 2022 and May 2023.

**
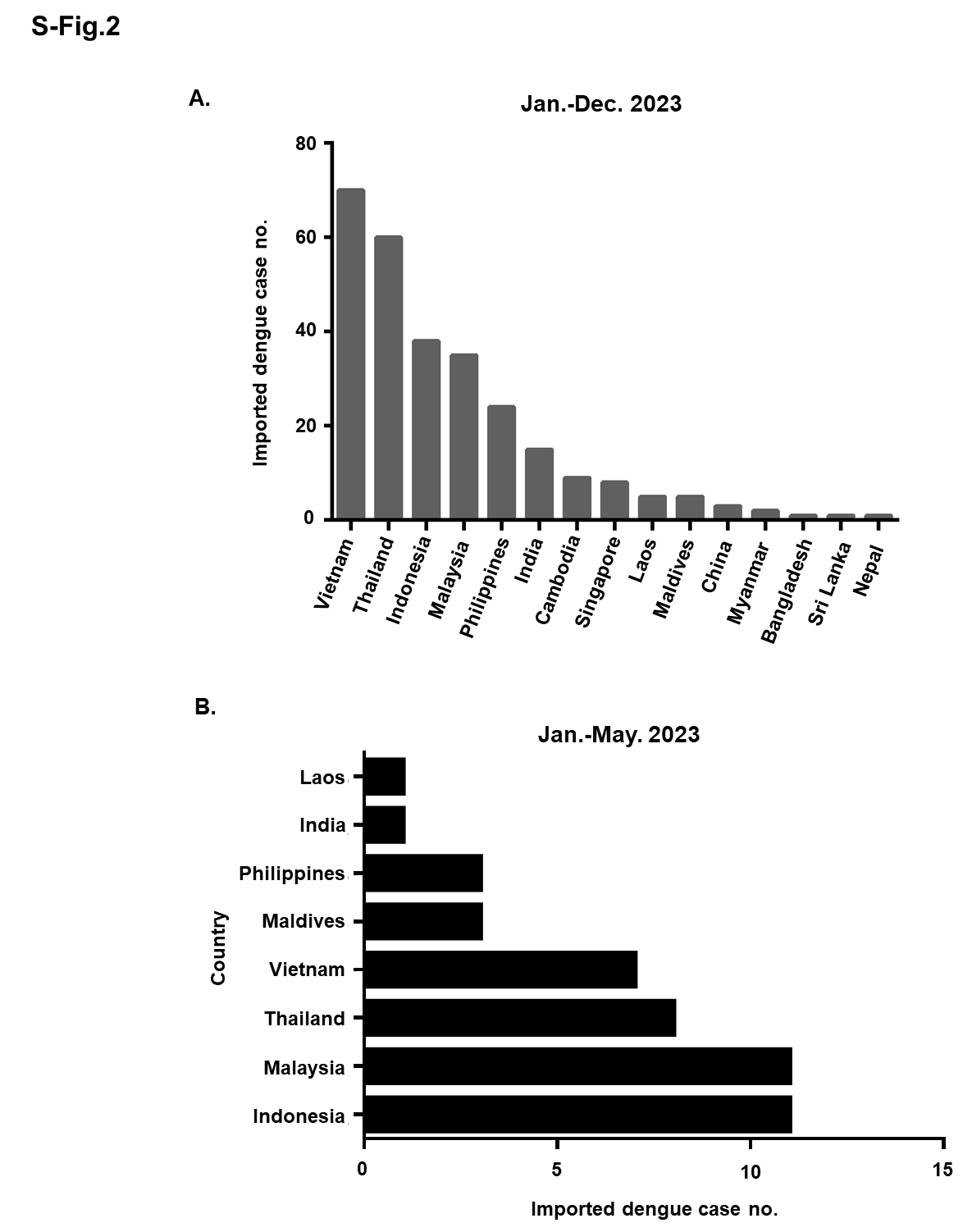
**

**S-Figure 2. Imported DENV cases associated with the 2023 outbreak by country of origin.** (A) Number of imported DENV cases reported from January to December 2023, categorized by country. (B) Number of imported DENV cases reported from January to May 2023, categorized by country. Data are presented as bar charts.

**
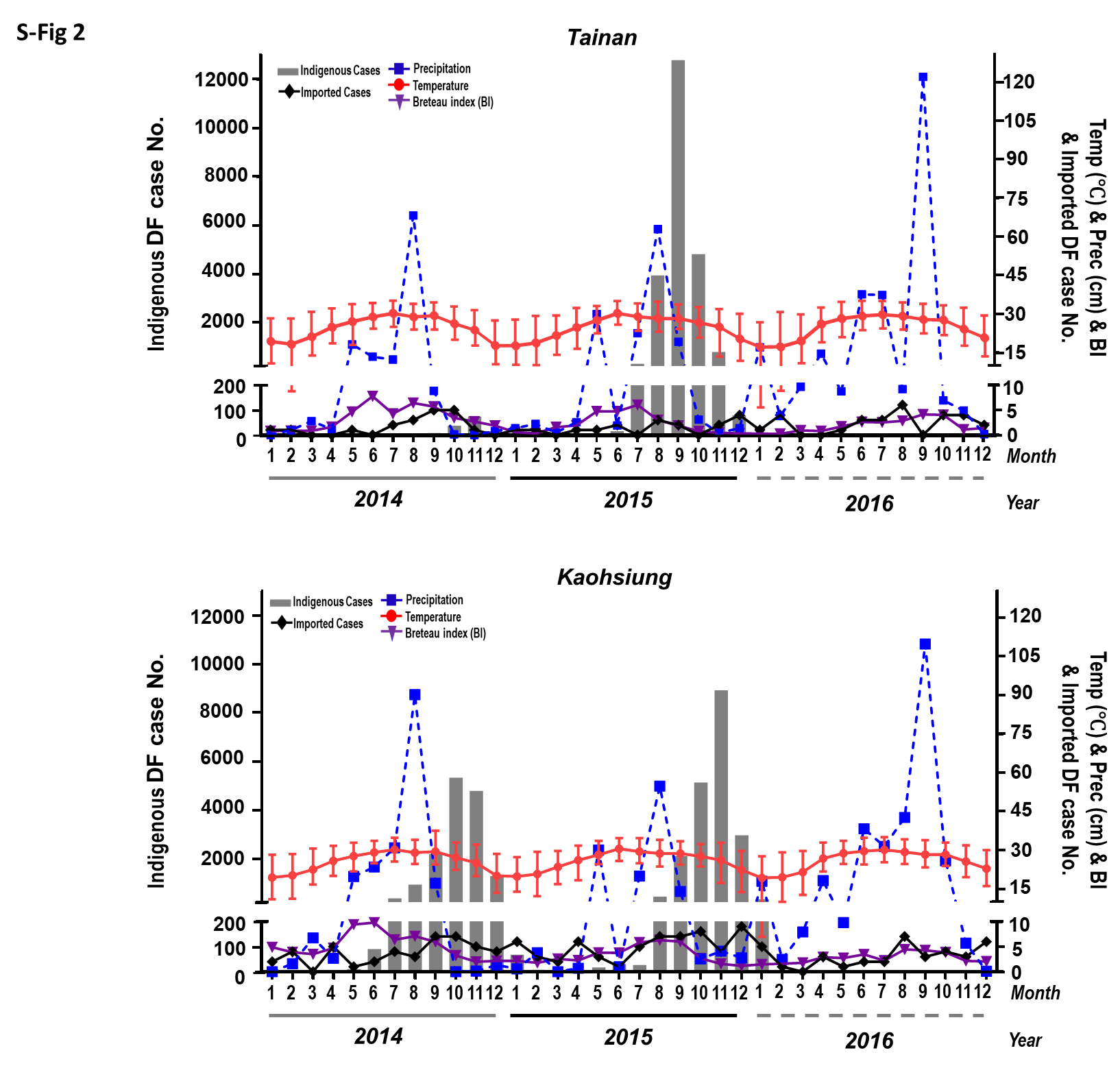
**

**S-Figure 3. Climatic variation, mosquito density, and imported cases associated with the past 2014–2015 dengue outbreaks in two major southern cities of Taiwan.**

(A) Tainan: Monthly indigenous and imported dengue cases during 2014–2016, shown together with ambient temperature, precipitation, and the Breteau Index (BI). (B) Kaohsiung: Monthly indigenous and imported dengue cases during 2014–2016, shown together with ambient temperature, precipitation, and the Breteau Index (BI). DF, dengue fever; Temp, temperature; Prec, precipitation; BI, Breteau Index.

**S-Table1. Information and GenBank accession numbers of dengue virus isolates**

| **Serotype** | **Strain/ID** | **Country** | **Year** | **Accession No.** |
| --- | --- | --- | --- | --- |
| DENV-1 | DV1/TW-KH-PX460799 2023 | Taiwan | 2023 | PX460799 |
| DENV-1 | DV1/TW-KH-PX460800 2023 | Taiwan | 2023 | PX460800 |
| DENV-1 | DV1/TW-KH-PX460801 2023 | Taiwan | 2023 | PX460801 |
| DENV-1 | DV1/TW-KH-PX460802 2023 | Taiwan | 2023 | PX460802 |
| DENV-1 | DV1/TW-KH-PX460803 2023 | Taiwan | 2023 | PX460803 |
| DENV-1 | DV1/TW-KH-PX460804 2023 | Taiwan | 2023 | PX460804 |
| DENV-1 | DV1/TW-KH-PX460805 2023 | Taiwan | 2023 | PX460805 |
| DENV-1 | DV1/TW-KH-PX460806 2023 | Taiwan | 2023 | PX460806 |
| DENV-1 | DV1/TW-KH-PX460807 2023 | Taiwan | 2023 | PX460807 |
| DENV-1 | DV1/TW-KH-PX460808 2023 | Taiwan | 2023 | PX460808 |
| DENV-1 | DV1/TW-KH-PX460809 2023 | Taiwan | 2023 | PX460809 |
| DENV-1 | DV1/TW-KH-PX460810 2023 | Taiwan | 2023 | PX460810 |
| DENV-1 | DV1/TW-KH-PX460811 2023 | Taiwan | 2023 | PX460811 |
| DENV-1 | DV1/TW-KH-PX460812 2023 | Taiwan | 2023 | PX460812 |
| DENV-1 | DV1/TW-KH-PX499695 2023 | Taiwan | 2023 | PX499695 |
| DENV-1 | DV1/TW-KH-PX499696 2023 | Taiwan | 2023 | PX499696 |
| DENV-1 | DV1/TW-KH-PX460990 2023 | Taiwan | 2023 | PX460990 |
| DENV-1 | DV1/TW-KH-PX499700 2014 | Taiwan | 2014 | PX499700 |
| DENV-1 | DV1/TW-KH-PX499701 2014 | Taiwan | 2014 | PX499701 |
| DENV-1 | DV1/TW-KH-PX499702 2014 | Taiwan | 2014 | PX499702 |
| DENV-2 | DV2/TW-KH-PX460823 2023 | Taiwan | 2023 | PX460823 |
| DENV-2 | DV2/TW-KH-PX460824 2023 | Taiwan | 2023 | PX460824 |
| DENV-2 | DV2/TW-KH-PX460825 2023 | Taiwan | 2023 | PX460825 |
| DENV-2 | DV2/TW-KH-PX460826 2023 | Taiwan | 2023 | PX460826 |
| DENV-2 | DV2/TW-KH-PX460872 2023 | Taiwan | 2023 | PX460827 |
| DENV-2 | DV2/TW-KH-PX460828 2023 | Taiwan | 2023 | PX460828 |
| DENV-2 | DV2/TW-KH-PX460829 2023 | Taiwan | 2023 | PX460829 |
| DENV-2 | DV2/TW-KH-PX460830 2023 | Taiwan | 2023 | PX460830 |
| DENV-2 | DV2/TW-KH-PX460831 2023 | Taiwan | 2023 | PX460831 |
| DENV-2 | DV2/TW-KH-PX460832 2023 | Taiwan | 2023 | PX460832 |
| DENV-2 | DV2/TW-KH-PX460833  2023 | Taiwan | 2023 | PX460833 |
| DENV-2 | DV2/TW-KH-PX460834 2023 | Taiwan | 2023 | PX460834 |
| DENV-2 | DV2/TW-KH-PX460835 2023 | Taiwan | 2023 | PX460835 |
| DENV-2 | DV2/TW-KH-PX460836 2023 | Taiwan | 2023 | PX460836 |
| DENV-2 | DV2/TW-KH-PX460837 2023 | Taiwan | 2023 | PX460837 |
| DENV-2 | DV2/TW-KH-PX460838 2023 | Taiwan | 2023 | PX460838 |
| DENV-2 | DV2/TW-KH-PX460918 2023 | Taiwan | 2023 | PX460918 |
| DENV-2 | DV2/TW-KH-PX499693 2023 | Taiwan | 2023 | PX499693 |
| DENV-2 | DV2/TW-KH-PX499694 2023 | Taiwan | 2023 | PX499694 |
| DENV-2 | DV2/TW-KH-PX499697 2015 | Taiwan | 2015 | PX499697 |
| DENV-2 | DV2/TW-KH-PX499698 2015 | Taiwan | 2015 | PX499698 |
| DENV-2 | DV2/TW-KH-PX499699 2015 | Taiwan | 2015 | PX499699 |

**S-Table 2. Reference dengue virus strains used for phylogenetic tree analysis**

| **Serotype** | **Strain/ID** | **Country** | **Year** | **Accession No.** |
| --- | --- | --- | --- | --- |
| DENV-1 | DV1/Malaysia/07211Y14/2014 | Malaysia | 2014 | KJ806963 |
| DENV-1 | DV1/Singapore/07771Y14/2014 | Singapore | 2014 | KJ806959 |
| DENV-1 | DV1/Singapore/41757Y13/2013 | Singapore | 2013 | KJ806949 |
| DENV-1 | DV1/Singapore/35765Y12/2012 | Singapore | 2012 | KJ806938 |
| DENV-1 | DV1/Malaysia/946Y14/2014 | Malaysia | 2014 | KJ806874 |
| DENV-1 | DV1/Malaysia/2008Y13/2013 | Malaysia | 2008 | KJ806848 |
| DENV-1 | DV1/Malaysia/643Y13/2013 | Malaysia | 2013 | KJ806836 |
| DENV-1 | DV1/Malaysia/858Y13/2013 | Malaysia | 2013 | KJ806828 |
| DENV-1 | DV1/Malaysia/2757Y13/2013 | Malaysia | 2013 | KJ806825 |
| DENV-1 | DV1/Malaysia/2779Y13/2013 | Malaysia | 2013 | KJ806824 |
| DENV-1 | DV1/Malaysia/5124Y12/2013 | Malaysia | 2013 | KJ806818 |
| DENV-1 | DV1/Malaysia/584Y12/2012 | Malaysia | 2012 | KJ806817 |
| DENV-1 | DV1/USA/HAWAII/1945 | USA | 1945 | AF425619 |
| DENV-1 | DV1/Nicaragua/CNM438VI03/2003 | Nicaragua | 2003 | DQ016657 |
| DENV-1 | DV1/India/WI52VI04/2003 | India | 2003 | DQ016653 |
| DENV-1 | DV1/India/055290/2005 | India | 2005 | JF297581 |
| DENV-1 | DV1/Jamaica/PRS 288690/1977 | Jamaica | 1977 | AF425621 |
| DENV-1 | DV1/Indonesia/SC01/2004 | Indonesia | 2004 | AY858983 |
| DENV-1 | DV1/Philippines/02SA029/2002 | Philippines | 2002 | AY422779 |
| DENV-1 | DV1/Australia/HATI7/1983 | Australia | 1983 | AF425612 |
| DENV-1 | DV1/Reunion/265/04/2004 | Reunion | 2004 | DQ285553 |
| DENV-1 | DV1/Laos/JH8/2011/2011 | Laos | 2011 | KF926700 |
| DENV-1 | DV1/China-Guangzhougi/11GZ12/2011 | China | 2011 | KC006944 |
| DENV-1 | DV1/China-Guangzhougi/GZ16/2006 | China | 2006 | EF113152 |
| DENV-1 | DV1/China-Guangzhougi/GZ01/95/1995 | China | 1995 | DQ855297 |
| DENV-1 | DV1/China/GZ1131/CHN/2002 | China | 2002 | JQ277840 |
| DENV-1 | DV1/Indonesia/D1/SBY95/10/2010 | Indonesia | 2010 | AB624554 |
| DENV-1 | DV1/Indonesia/0809aTw/2008 | Indonesia | 2008 | JF967832 |
| DENV-1 | DV1/Taiwan/248TP1010a/2010 | Taiwan | 2010 | JQ403520 |
| DENV-1 | DV1/Taiwan/511CH0909a/2010 | Taiwan | 2010 | JQ403519 |
| DENV-1 | DV1/Taiwan/718TN1008a/2010 | Taiwan | 2010 | JQ403521 |
| DENV-1 | DV1/Taiwan/111TP0808a/2008 | Taiwan | 2008 | JQ403516 |
| DENV-1 | DV1/Taiwan/811KH0807a/2008 | Taiwan | 2008 | JQ403517 |
| DENV-1 | DV1/Taiwan/806KH0810a/2008 | Taiwan | 2008 | JQ403518 |
| DENV-1 | DV1/Vietnam/1012aTw/2010 | Vietnam | 2010 | JF967953 |
| DENV-1 | DV1/Vietnam/0904bTw/2009 | Vietnam | 2009 | JF967859 |
| DENV-1 | DV1/Thailand/1010aTw/2010 | Thailand | 2010 | JF967940 |
| DENV-1 | DV1/Thailand/0810aTw/2008 | Thailand | 2008 | JF967838 |
| DENV-1 | DV1/Thailand/0910aTw/2009 | Thailand | 2009 | JF967888 |
| DENV-1 | DV1/Thailand/02-38-1HuNIID/2002 | Thailand | 2002 | AB111078 |
| DENV-1 | DV1/Thailand/01A00106/2001 | Thailand | 2001 | A00106 |
| DENV-1 | DV1/Philippines/1009aTw/2010 | Philippines | 2010 | JF967936 |
| DENV-1 | DV1/Philippines/1009bTw/2010 | Philippines | 2010 | JF967937 |
| DENV-1 | DV1/Philippines/1009cTw/2010 | Philippines | 2010 | JF967938 |
| DENV-1 | DV1/Philippines/2010 | Philippines | 2010 | JN415517 |
| DENV-1 | DV1/Malaysia/0811aTw/2008 | Malaysia | 2008 | JF967847 |
| DENV-1 | DV1/Malaysia/1008aTw/2010 | Malaysia | 2010 | JF967920 |
| DENV-1 | DV1/Cambodia/0807aTw/2008 | Cambodia | 2008 | JF967808 |
| DENV-1 | DV1/Taiwan/360052/1994 | Taiwan | 1994 | AB608789 |
| DENV-1 | DV1/Taiwan/832/1994 | Taiwan | 1994 | AB608788 |
| DENV-1 | DV1/Taiwan/NDF30/2008 | Taiwan | 2008 | AB608786 |
| DENV-1 | DV1/Indonesia/SBY163/2013 | Indonesia | 2013 | AB915380 |
| DENV-1 | DV1/Thalaind/TM19/2019 | Thalaind | 2019 | MZ619041 |
| DENV-1 | DV1/Th18-098/Thailand/2018 | Thailand | 2018 | MN955660 |
| DENV-1 | DV1/JX30/Nanchang/2019 | Nanchang | 2019 | MN894251 |
| DENV-1 | DV1/Th18-071/Thailand/2018 | Thailand | 2018 | MN955650 |
| DENV-1 | DV1/11940/Lao/2019 | Lao | 2019 | MW559405 |
| DENV-1 | DV1/16197/Lao/2020 | Lao | 2020 | MW559413 |
| DENV-1 | DV1/23GZ20098/Huizhou/2023 | Huizhou | 2023 | GZ20098 |
| DENV-1 | DV1/SUB-049A/Indonesia/2018 | Indonesia | 2018 | KY057371 |
| DENV-1 | DV1/JMB20-074/Indonesia/2020 | Indonesia | 2020 | OP198507 |
| DENV-1 | DV1/Taiwan/806KH1405a/2016 | Taiwan | 2016 | KU365900 |
| DENV-1 | DV1/Taiwan/709TN0706a/2017 | Taiwan | 2017 | KX951690 |
| DENV-1 | DV1/Taiwan/114TP1611a/2017 | Taiwan | 2017 | KY496854 |
| DENV-1 | DV1/Taiwan/813KH9809b/2017 | Taiwan | 2017 | KY496856 |
| DENV-1 | DV1/Thailand/2023 | Thailand | 2023 | PV344272 |
| DENV-1 | DV1/Thailand/2023 | Thailand | 2023 | PV663083 |
| DENV-1 | DV1/Thaialnd/2023 | Thaialnd | 2023 | PV663088 |
| DENV-1 | DV1/Taiwan/717TN2306a/2023_Outbreak | Taiwan | 2023 | PP087791 |
| DENV-1 | DV1/Taiwan/646YU2306a/2023_Outbreak | Taiwan | 2023 | PP087792 |
| DENV-1 | DV1/SM1/Yunnan/2023 | Yunnan | 2023 | PP396094 |
| DENV-1 | DV1/SM2/Yunnan/2023 | Yunnan | 2023 | PP396095 |
| DENV-1 | DV1/ChinaZj/yw01/2013/KF864667-G5 | ChinaZj | 2013 | KF864667 |
| DENV-1 | DV1/IN/RGCB592/2009-G5 | India | 2009 | JN903581 |
| DENV-1 | DV1/SG(EHI)DED65008/Singapore/2008 | Singapore | 2008 | GQ357692 |
| DENV-1 | DV1/055290/India/2005 | India | 2005 | F297581 |
| DENV-1 | DV1/98901530/Indonesia/1998 | Indonesia | 1998 | AB189121 |
| DENV-1 | DV1/GD03/China/1991 | China | 1991 | FJ196845 |
| DENV-1 | DV1/HCS1/AUS/1983 | AUS | 1983 | AF425611 |
| DENV-1 | DV1/Malaysia/36046/2005 | Malaysia | 2005 | FN825674 |
| DENV-1 | DV1/Malaysia/P72-1244/1972-G3 | Malaysia | 1972 | AF425622 |
| DENV-1 | DV1/16007/Thailabd/1964 | Thailabd | 1964 | AF180817 |
| DENV-1 | DV1/606147/Thaialnd/1960 | Thaialnd | 1960 | JF297570 |
| DENV-1 | DV1/P72-1244/EF457905-G3 | Malaysia | 1972 | EF457905 |
| DENV-1 | DV1/Malaysia/36046/05 | Malaysia | 2005 | FN825674 |
| DENV-1 | DV1/Myanmar/M49440/KF559254-G4 | Myanmar | 2002 | M49440 |
| DENV-1 | DV1/Philiphones/2010/KR919811-G4 | Philiphones | 2010 | KR919811 |
| DENV-1 | DV1/Indonesia/2014/KR919812-G4 | Indonesia | 2014 | KR919812 |
| DENV-1 | DV1/Papua New Guinea/PNG2014a/KR919814-G4 | Papua New Guinea | 2014 | KR919814 |
| DENV-1 | DV1/IND/715393/1971/JQ922546-G5 | India | 1971 | JQ922546 |
| DENV-1 | DV1/India/2005/JF297581-G5 | India | 2005 | JF297581 |
| DENV-1 | DV1/India/1962/JF297572-G5 | India | 1962 | JF297572 |
| DENV-1 | DV1/Paraguary/2000/AF514883-G5 | Paraguay | 2000 | AF514883 |
| DENV-1 | DV1/BR/BID-V3490/2008/GU131863-G5 | BR | 2008 | GU131863 |
| DENV-1 | DV1/Brunei/KR919820/2014-G6 | Brunei | 2014 | KR919820 |
| DENV-1 | DV1/Thialand/1963/AF425629-G2 | Thailand | 1963 | AF425629 |
| DENV-1 | DV1/Thaialnd/1954/D10513-G2 | Thailand | 1954 | D10513 |
| DENV-2 | D2/Indonesia/1001aTw/2010 | Indonesia | 2010 | JF968003 |
| DENV-2 | D2/Indonesia/1003aTw/2010 | Indonesia | 2010 | JF968008 |
| DENV-2 | D2/Indonesia/1007aTw/2010 | Indonesia | 2010 | JF968022 |
| DENV-2 | D2/Indonesia/1012aTw/2010 | Indonesia | 2010 | JF968050 |
| DENV-2 | D2/India/1009aTw/2010 | India | 2010 | JF968040 |
| DENV-2 | D2/Philippines/1004aTw/2010 | Philippines | 2010 | JF968011 |
| DENV-2 | D2/Philippines/1009aTw/2010 | Philippines | 2010 | JF968037 |
| DENV-2 | D2/Philippines/1011aTw/2010 | Philippines | 2010 | JF968046 |
| DENV-2 | D2/Philippines/0308aTw | Philippines | 2003 | EU448418 |
| DENV-2 | D2/NewGuinea/1944 | NewGuinea | 1944 | M29095 |
| DENV-2 | D2/Malaysia/1002aTw/2010 | Malaysia | 2010 | JF968005 |
| DENV-2 | D2/Malaysia/1008aTw/2010 | Malaysia | 2010 | JF968035 |
| DENV-2 | D2/Malaysia/1011aTw/2010 | Malaysia | 2010 | JF968049 |
| DENV-2 | D2/Malaysia/01954Y14/2014 | Malaysia | 2014 | KJ806810 |
| DENV-2 | D2/Malaysia/05400Y14/2014 | Malaysia | 2014 | KJ806812 |
| DENV-2 | D2/Malaysia/1451Y13/2013 | Malaysia | 2013 | KJ806921 |
| DENV-2 | D2/Malaysia/4544Y13/2013 | Malaysia | 2013 | KJ806919 |
| DENV-2 | D2/Malaysia/681Y13/2013 | Malaysia | 2013 | KJ806886 |
| DENV-2 | D2/Malaysia/85Y12/2012 | Malaysia | 2012 | KJ806878 |
| DENV-2 | D2/Malaysia/708Y13/2013 | Malaysia | 2013 | KJ806896 |
| DENV-2 | D2/Malaysia/3576Y12/2012 | Malaysia | 2012 | KJ806877 |
| DENV-2 | D2/Malaysia/535Y13/2013 | Malaysia | 2013 | KJ806882 |
| DENV-2 | D2/Malaysia/3514Y13/2013 | Malaysia | 2013 | KJ806889 |
| DENV-2 | D2/Thailand/1004aTw/2010 | Thailand | 2010 | JF968013 |
| DENV-2 | D2/Thailand/1007aTw/2010 | Thailand | 2010 | JF968029 |
| DENV-2 | D2/Thailand/1010aTw/2010 | Thailand | 2010 | JF968045 |
| DENV-2 | D2/Vietnam/1006aTw/2010 | Vietnam | 2010 | JF968019 |
| DENV-2 | D2/Vietnam/1008aTw/2010 | Vietnam | 2010 | JF968027 |
| DENV-2 | D2/Vietnam/1012aTw/2010 | Vietnam | 2010 | JF968052 |
| DENV-2 | D2/Laos/1006aTw/2010 | Laos | 2010 | JF968020 |
| DENV-2 | D2/Singapore/1007aTw/2010 | Singapore | 2010 | JF968025 |
| DENV-2 | D2/Singapore/1008aTw/2010 | Singapore | 2010 | JF968034 |
| DENV-2 | D2/Singapore/1012aTw/2010 | Singapore | 2010 | JF968051 |
| DENV-2 | D2/Myanmar/1007aTw/2010 | Myanmar | 2010 | JF968026 |
| DENV-2 | D2/Taiwan/830KH0809a/2008 | Taiwan | 2008 | JQ403522 |
| DENV-2 | D2/Taiwan/900PT0910a/2009 | Taiwan | 2009 | JQ403523 |
| DENV-2 | D2/Taiwan/807KH1008a/2010 | Taiwan | 2010 | JQ403524 |
| DENV-2 | D2/China/14D2GDZS00F/2014 | China | 2014 | KP064521 |
| DENV-2 | D2/China/GZ25/2014 | China | 2014 | KP723478 |
| DENV-2 | D2/China/14D2GDZS00B/2014 | China | 2014 | KP064518 |
| DENV-2 | D2/TN-1711-2015-TW | Taiwan | 2015 | MH051830 |
| DENV-2 | D2/TN-1637-2015-TW | Taiwan | 2015 | MH051829 |
| DENV-2 | D2/KH-411-2015-TW | Taiwan | 2015 | MH051816 |
| DENV-2 | D2/KH-271-2015-TW | Taiwan | 2015 | MH051819 |
| DENV-2 | D2/Singapore/07664Y12/2012 | Singapore | 2012 | KJ806775 |
| DENV-2 | D2/Singapore/34994Y13/2013 | Singapore | 2013 | KJ806785 |
| DENV-2 | D2/Singapore/40590Y13/2013 | Singapore | 2013 | KJ806791 |
| DENV-2 | D2/Malaysia/02803Y14/2014 | Malaysia | 2014 | KJ806811 |
| DENV-2 | D2/Pakistan/I1/2014 | Pakistan | 2014 | KT239353 |
| DENV-2 | D2/Pakistan/I2/2014 | Pakistan | 2014 | KT239354 |
| DENV-2 | D2/Pakistan/I3/2014 | Pakistan | 2014 | KT239355 |
| DENV-2 | D2/Indonesia/Hu/NIID24/2015 | Indonesia | 2015 | LC064746 |
| DENV-2 | D2/109-0003/Cambodia/2019 | Cambodia | 2019 | OL414730 |
| DENV-2 | D2/100-0670/Cambodia/2019 | Cambodia | 2019 | OL412740 |
| DENV-2 | D2/Myseq60/Shanghai/2019 | Shanghai | 2019 | MT856323 |
| DENV-2 | D2/109-0292/Cambodia/2020 | Cambodia | 2020 | OL414746 |
| DENV-2 | D2/CN/HZ19-71/2019 | China | 2019 | OP684216 |
| DENV-2 | D2/C-TM19-46/Thailand/2019 | Thailand | 2019 | MZ636781 |
| DENV-2 | D2/TW/813KH2307a/2023 | TW | 2023 | PP087793 |
| DENV-2 | DV2/Laos/2023 | Laos | 2023 | PQ775639 |
| DENV-2 | DV2/Laos/2022 | Laos | 2022 | PQ775622 |
| DENV-2 | DV2/Laos/2023 | Laos | 2023 | PQ775625 |
| DENV-2 | DV2/Thailand/2024 | Thailand | 2024 | PV663123 |
| DENV-2 | DV2/Shanghai-China/2019 | China | 2019 | MT856323 |
| DENV-2 | DV2/Laos/2022 | Laos | 2022 | PQ775623 |
| DENV-2 | D2/Mexico/131/1992 | Mexico | 1992 | AF100469 |
| DENV-2 | D2/Colombia/I348600/1996 | Colombia | 1996 | I348600 |
| DENV-2 | D2/Senegal/DAKHD10674/1970 | Senegal | 1970 | HD10674 |
| DENV-2 | D2/IPC/BID-V3922/Combodia/2008 | Cambodia | 2008 | GU131924 |
| DENV-2 | D2/DR59/01 | Dominican Republic | 2004 | AB122022 |
| DENV-2 | D2/St Vincent/1009aTw | Saint Vincent and the Grenadines | 2010 | JF968043 |
| DENV-2 | D2/BR0690/RJ/2008 | Brazil | 2008 | HQ026763 |
| DENV-2 | D2/0294/Cambodia/2020 | Cambodia | 2020 | OL414736 |

**S-Table 3. Dengue Fever in Taiwan during 2013-2023**

| **Year** | **DENV cases** |  | **Identified DENV Strains** | | **Country of Importation** |
| --- | --- | --- | --- | --- | --- |
|  | **Imported Indigenous** |  | **Serotype** | **Genotype** |  |
| 2013 | 261 596 |  | DENV-1 | Genotype I | Dominican /Malaysia |
|  |  |  | DENV-2* | Cosmopolitan | Indonesia |
|  |  |  | DENV-3 | Genotype I | Indonesia |
| 2014 | 245 15509 |  | DENV-1* | Genotype I | Indonesia/ Philippines |
| 2015 | 365 43418 |  | DENV-1* | Genotype I | Indonesia/Malaysia/China |
|  |  |  | DENV-2* | Cosmopolitan | Indonesia/China |
| 2016 | 363 380 |  | DENV-1 | Genotype I | Indonesia/Malaysia |
|  |  |  | DENV-2* | Cosmopolitan | Indonesia/Philippine |
| 2017 | 333 10 |  | DENV-1 | Genotype I | Southeast Asia |
|  |  |  | DENV-3 | Genotype I | Philippines/Indonesia |
| 2018 | 350 183 |  | DENV-1 | Genotype I | Vietnam/Philippines |
|  |  |  | DENV-2 | Cosmopolitan | Philippines/Thailand |
| 2019 | 540 100 |  | DENV-1 | Genotype I | Indonesia |
|  |  |  | DENV-2* | Cosmopolitan | Vietnam/Philippines |
|  |  |  | DENV-3 | Genotype I | Philippines |
|  |  |  | DENV-4* | Genotype II | Indonesia |
| 2020 | 64 73 |  | DENV-1 | Genotype I | Indonesia |
| 2021 | 12 0 |  | NA |  | NA |
| 2022 | 68 20 |  | DENV-1* | Genotype I | Indonesia/Thailand |
| 2023 | 277 26706 |  | DENV-1* | Genotype I | Indonesia/Thailand/Lao/China |
|  |  |  | DENV-2* | Cosmopolitan | Thailand/ China/ Cambodia |

Footnote: Data were obtained from annual surveillance reports of the Taiwan Centers for Disease Control (CDC). Indigenous and imported cases were classified based on travel history within 14 days prior to symptom onset. Severe dengue (SD) and dengue fever (DF) were defined according to Taiwan CDC/WHO 2009 guidelines.

**S-Table 4. Three Dengue Outbreaks in Taiwan Over the Past Decade**

| **Year** | **DF No.** | **SD No.** | **Death No.** | **SD** **CFR(%)** |
| --- | --- | --- | --- | --- |
| **2014** | 15732 | 136 | 21 | 15.4% |
| **2015** | 43784 | 647 | 158 | 24.4% |
| **2023** | 26706 | 137 | 44 | 32.1% |

Footnote: The three major dengue outbreaks in Taiwan occurred in 2014, 2015, and 2023. Case numbers were obtained from the Taiwan Centers for Disease Control (CDC), and all dengue virus (DENV) infections were laboratory confirmed. Abbreviations: DF – dengue fever; SD – severe dengue; CFR – case fatality rate.

**S-Table 5. Flowchart of Patient Selection for Dengue Virus Serotyping**

| **Selection steps** | **Cases No. (%)** | |
| --- | --- | --- |
| 1. Dengue-suspected patients enrolled in this study | | 1276 |
| 2.DENV RT-PCR–positive cases | | 206 (16.1%, 206/1276) |
| 3. Randomly selected cases for molecular serotyping | | 1. (53.6 %, 116/206) |
| 4. Molecular serotyping results: | |  |
| ─ *DENV-1* | | 60 (51.7%, 60/116) |
| ─ *DENV-2* | | 56 (48.3%, 58/116) |
| ─ *DENV-3* | | 0 (0%) |
| ─ *DENV-4* | | 0 (0%) |
| 5. Virus culture positive  *-DENV-1*  *-DENV-2* | | 81 (69.8%, 81/116)  45 (55.6% 45/81)  36 (44.4%, 36/81) |
|  | |  |

Footnote: All cases included in this summary were initially reported as suspected dengue cases during the 2023 outbreak in Taiwan. Serotyping was performed using quantitative real-time RT-PCR.
